# Effect of Combined Iron Oxide Magnetic Nanoparticles and Magnetic Field Exposure on Osteogenic Differentiation of Mesenchymal Stem Cells: A Systematic Review and Meta-Analysis

**DOI:** 10.3390/bioengineering13101143

**Published:** 2026-09-30

**Authors:** Maksim Solopov, Roman Ishchenko, Viktor Turchin, Viktoria Bushe, Anna Kavelina, Gulnara Akopian, Elizaveta Lebed, Andrey Popandopulo

**Affiliations:** V.K. Gusak Institute of Emergency and Reconstructive Surgery, 283045 Donetsk, Russia

**Keywords:** mesenchymal stem cells, magnetic nanoparticles, iron oxide, magnetic field, osteogenic differentiation, bone tissue engineering

## Abstract

Combined iron oxide magnetic nanoparticles (MNPs) and external magnetic fields (MFs) are explored to direct osteogenic differentiation of mesenchymal stem cells (MSCs) in bone tissue engineering, yet exposure parameters vary widely. This systematic review and meta-analysis searched PubMed and Dimensions, with Consensus and SciSpace as exploratory scoping aids (final search 22 November 2025). Twenty-five studies entered qualitative synthesis and twenty-two quantitative synthesis (‘MNP + MF’ vs. ‘MNP without MF’; standardized mean difference [SMD] as Hedges’ g; restricted maximum likelihood random-effects model with Hartung–Knapp–Sidik–Jonkman confidence intervals [CIs]). Across nine outcomes, effect directions were predominantly positive; uncorrected significance held only for alkaline phosphatase (ALP) activity (SMD = +4.42; 95% CI 0.97–7.86; k = 14) and mineralization (SMD = +2.41; 0.03–4.80; k = 7). After Holm correction, no outcome remained significant. Heterogeneity was critical (I^2^ > 75% for five outcomes). Funnel–plot asymmetry for ALP suggested possible small-study effects; trim-and-fill reduced the pooled SMD to +1.34 (−1.60–4.27). Findings constitute a low-certainty, hypothesis-generating signal rather than confirmed efficacy; standardized protocols and blinded designs are needed before translational claims.

## 1. Introduction

Restoration of critical-sized bone defects remains one of the unresolved challenges of regenerative medicine [1]. Cell-based technologies utilizing mesenchymal stem cells (MSCs) are considered the cornerstone of bone tissue engineering owing to the capacity of these cells for osteogenic differentiation [2,3,4]. A key challenge is the identification of safe and controllable methods for directed stimulation of osteogenesis that do not require genetic modification or continuous presence of growth factors [5,6,7,8].

Magnetic nanoparticles (MNPs) based on iron oxides (predominantly magnetite Fe_3_O_4_ and maghemite γ-Fe_2_O_3_), including superparamagnetic iron oxide nanoparticles (SPIONs), possess biocompatibility, the ability to be internalized by cells, and responsiveness to external magnetic fields (MFs) [9,10]. Upon application of a static, alternating, or pulsed MF to cells loaded with MNPs or residing within a magnetically active matrix, local mechanical forces arise [11,12]. These forces are transmitted to the cytoskeleton and mechanosensitive structures such as integrins and ion channels, including the TWIK-related potassium channel-1 (TREK-1) [12,13], thereby activating mechanotransduction cascades (e.g., Yes-associated protein/transcriptional coactivator with PDZ-binding motif (YAP/TAZ) [14,15], mitogen-activated protein kinase (MAPK) [16,17], bone morphogenetic protein (BMP)/Smad [18], Wnt [19,20]) and are capable of enhancing osteogenic differentiation.

The aim of the present systematic review and meta-analysis was to summarize and quantitatively evaluate the effect of combined magnetic field exposure on MSCs associated with MNPs on the expression of key markers of osteogenic differentiation, to explore potentially influential exposure characteristics, and to identify sources of heterogeneity that may influence effect magnitudes. By synthesizing the available pre-clinical evidence, this review is intended to inform the design of magnetically responsive biomaterials and non-invasive physical stimulation strategies in bone tissue engineering, and to provide the broader scientific community with a transparent appraisal of what can—and cannot—currently be concluded about optimal magnetic regimens. Because static, low-frequency alternating, and pulsed electromagnetic fields represent distinct physical regimes (radiofrequency hyperthermia being a further, thermal regime), any single summary estimate pooling all field types is treated throughout as an exploratory composite model; primary scientific interpretation is directed to the stratified subgroups.

## 2. Materials and Methods

This review was conducted in accordance with the Preferred Reporting Items for Systematic Reviews and Meta-Analyses (PRISMA) 2020 recommendations [21]. The protocol is provided in the Supplementary Materials (File S1). The protocol was registered retrospectively on the Open Science Framework platform (https://osf.io/5kwmx/). The research question together with the inclusion criteria were formulated according to the PICOS framework [22].

### 2.1. Search Sources and Strategy

Literature searches were conducted in primary bibliographic databases (PubMed, Dimensions). AI-powered literature discovery platforms Consensus and SciSpace were used solely as exploratory scoping aids to surface potentially relevant records via natural-language queries; they are not conventional bibliographic databases, rely on proprietary non-deterministic semantic ranking, and therefore cannot satisfy PRISMA 2020 reproducibility requirements for formal database searching. They were added because terminology in this interdisciplinary field (materials science, magnetism, and stem-cell biology) is heterogeneous, and semantic full-text retrieval can surface experimental reports that keyword-based Boolean queries may miss; all records they returned were screened against the same eligibility criteria. Their outputs were cross-checked against PubMed/Dimensions exports and did not replace structured Boolean searches. Limiting conventional database coverage to PubMed and Dimensions may have missed studies indexed only in Scopus, Web of Science, Embase, or IEEE Xplore; this bibliographic selection bias is acknowledged as a limitation. The final searches were completed on 22 November 2025 without date re-strictions. The search strategy employed a combination of controlled Medical Subject Headings (MeSH) terms and free-text keywords connected with Boolean AND and OR operators. Search queries comprised four thematic blocks: (1) mesenchymal stem cells (MSC) and source-specific terms such as adipose-derived stem cells (ADSC), bone marrow stromal cells (BMSC), and dental pulp stem cells (DPSC); (2) magnetic nano-particles (magnetite nanoparticles, iron oxide, Fe_3_O_4_, SPION); (3) magnetic exposures (magnetic fields, electromagnetic fields, static magnetic field (SMF), pulsed electro-magnetic field (PEMF)); (4) osteogenic differentiation and its markers (osteogenesis, osteogenic differentiation, alkaline phosphatase (ALP), alizarin red, runt-related transcription factor 2 (Runx2), osteocalcin, osterix, type I collagen (COL1/COL1A1), etc.). In addition, backward citation chasing (manual screening of reference lists of included studies and relevant reviews) was performed to ensure literature saturation. Detailed search queries and database export formats are provided in Supplementary Materials (Table S1).

### 2.2. Inclusion Criteria (PICOS)

Original experimental studies in which MSCs were associated with iron oxide-based MNPs and exposed to an external magnetic field in the presence of a comparable control group were included for qualitative synthesis. For quantitative synthesis (meta-analysis), studies were required to provide a comparator in which MNP exposure was present but magnetic-field exposure was absent (‘MNP + MF’ vs. ‘MNP without MF’), thereby isolating the specific contribution of the magnetic field. The influence on markers of osteogenic differentiation was assessed. Criteria were applied sequentially during title/abstract screening and full-text assessment; disagreements between reviewers were resolved by discussion. A summary of the inclusion and exclusion criteria is presented in Table 1.

### 2.3. Study Selection and Data Extraction

Duplicate records were removed with a custom Python script (Python v3.13; matching on DOI, PMID, and normalized title; code available from the corresponding author), after which records were managed in the reference manager Zotero v9.0.5 (Corporation for Digital Scholarship, Fairfax, VA, USA). Subsequently, two independent investigators performed title/abstract screening and full-text assessment; disagreements were resolved by discussion. Data were extracted into a standardized table organized in four categories: study and intervention characteristics, experimental group descriptions, outcome data, and calculated effect sizes. When data were presented only in graphical form, values were extracted using ImageJ v1.54p (National Institutes of Health, Bethesda, MD, USA) [23]: plot axes were calibrated against labeled tick marks, and the positions of means and error-bar limits were measured and converted to data units. In the absence of a reported number of replicates (*n*), a minimum value of *n* = 3 was assumed (conservative imputation), with a dedicated sensitivity analysis comparing pooled estimates with and without imputed studies (Section 3.8). Data extraction was performed independently by two investigators using the standardized form; discrepancies were resolved by discussion. No unpublished outcome data were used; data were obtained from the published reports and their Supplementary Materials.

To prevent the unit-of-analysis error that arises from repeated use of a single control group or correlated measurements, leading to artificial deflation of the standard error, exactly one comparison per study per outcome was extracted. Selection was performed according to pre-specified rules: (1) the key/standard time point of differentiation for a given marker was extracted (e.g., day 7 for ALP activity and mRNA expression; days 14–21 for mineralization and osteocalcin); (2) the primary working non-toxic MNP concentration (10–50 µg/mL) was selected; (3) the main magnetic stimulation regimen declared by the authors was chosen. Selection was based on pre-specified criteria rather than on the magnitude of the observed effect, thereby reducing the risk of outcome selection based on effect size. If only a single time point was available, it was used. The selected time points and groups were recorded in the data extraction table (see Supplementary Materials).

### 2.4. Risk of Bias Assessment and Certainty of Evidence

Risk of bias was assessed using the Office of Health Assessment and Translation (OHAT) Risk of Bias Tool developed by the National Toxicology Program (NTP/NIEHS) [24], adapted for preclinical in vitro cell culture studies. Because the original OHAT framework was formulated primarily for human observational and in vivo animal studies, we adapted its criteria across seven domains relevant to experimental cell biology: (1) randomization of sample/vessel allocation, (2) identical experimental conditions and personnel blinding, (3) outcome assessment blinding, (4) completeness of outcome data, (5) selective outcome reporting, (6) nanoparticle characterization and cytotoxicity control (verifying non-toxic working concentrations), and (7) experimental replication (independent biological replicates). Each domain was classified as ‘definitely low’, ‘probably low’, ‘probably high’, or ‘definitely high’ risk of bias according to predefined operational rules. In line with conservative preclinical evaluation standards, when key procedural safeguards (such as sample randomization or personal blinding) were not explicitly described, the respective domain was classified as ‘probably high risk of bias’ due to insufficient reporting, rather than assuming methodological compliance. Risk-of-bias assessments were performed independently by two investigators, with disagreements resolved by discussion.

The overall certainty of evidence for each primary outcome was evaluated using a predefined adaptation of the Grading of Recommendations Assessment, Development and Evaluation (GRADE) framework [25]. Because standard GRADE was established for clinical healthcare interventions, the initial certainty for controlled laboratory experimental studies was set at ‘high’ by analogy to randomized controlled trials given the tight experimental control over non-intervention variables, followed by structured downgrading across five standard domains: risk of bias, heterogeneity/inconsistency, indirectness, imprecision, and publication/reporting bias.

### 2.5. Data Synthesis and Statistical Analysis

For the quantitative synthesis, the comparison of ‘MNP + MF’ versus ‘MNP without MF’ groups was selected, enabling isolation of the magnetic field contribution. From each study, means, standard deviations, and sample sizes were extracted. Due to heterogeneous measurement units, the effect was expressed as the standardized mean difference (SMD) in the form of Hedges’ *g* (with the *J* correction factor that eliminates upward bias at small *n*) [26]:

(1) $$g=J\cdot \frac{\bar{X_{MNP+MF}}-\bar{X_{MNP\;without\;MF}}}{s_{pool}},$$

(2) $$s_{pool}=\sqrt{\frac{\left(n_{1}-1\right)s_{1}^{2}+(n_{2}-1)s_{2}^{2}}{n_{1}+n_{2}-2}},$$

(3) $$J=1-\frac{3}{4\left(n_{1}+n_{2}-2\right)-1},$$

where *X_MNP+MF_* and *X_MNP without MF_* are the mean values of the measured outcome in the experimental and control groups, respectively; *n*_1_ and *n*_2_ are the sample sizes in the experimental and control groups, respectively; *s*_1_ and *s*_2_ are the standard deviations in the experimental and control groups, respectively.

Positive SMD values correspond to enhancement of osteogenesis in the ‘MNP + MF’ group. According to Cohen’s benchmarks (the conventional scale for evaluating SMD magnitude), the effect was considered: ≈0.2—small, ≈0.5—medium, ≥0.8—large. For osteocalcin, type I collagen, and osteopontin, mRNA expression levels (RT-qPCR) and protein levels (immunoassay) were analyzed in separate pools. Similarly, alkaline phosphatase was evaluated as two distinct outcomes: ALP enzyme catalytic activity (spectrophotometric/fluorimetric substrate cleavage assays) and ALP mRNA expression. All studies pooled under ALP activity evaluated enzyme activity normalized to total protein or cell count; no immunoassay-based ALP protein abundance measurements were pooled. This separation is biologically essential because transcription and enzymatic/protein synthesis reflect different stages of osteogenesis and are measured at different time points. As a robustness check against inflated SMDs arising from near-zero within-group standard deviations typical of tightly controlled in vitro assays, we additionally computed the natural logarithm of the response ratio (lnRR) with delta-method variances [27] and re-pooled effects under the same random-effects model (Section 3.8).

Quantitative pooling of individual effect estimates within a specific outcome was performed using a random-effects model, which allows for variation in true effects across studies. Between-study variance *τ*^2^ was estimated by restricted maximum likelihood (REML) [28]. The pooled Hedges’ *g* estimate and its 95% confidence interval were calculated using the Hartung–Knapp–Sidik–Jonkman (HKSJ) method [29,30] with a t-distribution and *k* − 1 degrees of freedom (*k* = number of studies), which yields conservative intervals at small *k*. The presence and degree of heterogeneity were assessed using Cochran’s *Q* statistic and the *I*^2^ index [31]:

(4) $$I^{2}=\max\left(0,\frac{Q-df}{Q}\right)\cdot 100\%,$$

where *df* = *k* − 1. Cochran’s *Q* statistic is the weighted sum of squared deviations in individual effects *g_i_* from the pooled estimate *ḡ*:

(5) $$Q=\sum w_{i}{\left(g_{i}-\bar{g}\right)}^{2},$$

where *w_i_* is the inverse variance weight.

A statistically significant *Q* test (at the threshold *p_Q_* < 0.10, accounting for the low power of the test at small *k*) indicates evidence of heterogeneity beyond that expected from sampling error alone. *I*^2^ below 25% was considered low heterogeneity, 25–50%—moderate, 50–75%—substantial, and above 75%—high. When *I*^2^ > 75%, the pooled estimate was retained as an exploratory summary, but the primary emphasis shifted to narrative synthesis and analysis of more homogeneous subgroups. When *k* > 2, the 95% prediction interval (PI) was additionally calculated, indicating how unpredictable the effect would be in a new similar study given the existing heterogeneity.

In addition, subgroup analyses (by MF type, MSC source, magnetic flux density level), univariate meta-regression, leave-one-out sensitivity analysis, outlier-truncated sensitivity analysis (excluding comparisons with |SMD| > 10), sensitivity analysis excluding studies with imputed sample size, trim-and-fill adjustment for funnel–plot asymmetry, and Holm correction for nine primary outcomes were performed [32,33]. Funnel–plot asymmetry (small-study effects, including possible publication bias) was assessed using Egger’s regression test (regression of standardized effect on precision at *k* ≥ 10) [32]. Single summary estimates that pool static, alternating/time-varying, and pulsed fields are reported only as exploratory composite models. Statistical significance was not equated with biological or clinical significance. The *p*-value addressed the probability of a chance difference between groups; SMD was used to assess the direction and relative magnitude of the in vitro effect. A large SMD did not directly imply that the described effect was biologically meaningful or clinically applicable, as no established threshold for such significance exists for in vitro osteogenic markers. Calculations were implemented in Python v3.13 using the NumPy v2.4.6, SciPy, v1.16.3 and Matplotlib v3.11.0 libraries.

## 3. Results

### 3.1. Study Selection

The literature search identified a total of 191 records: PubMed (*n* = 26), Dimensions (*n* = 64), Consensus (*n* = 13), and SciSpace (*n* = 88). Following removal of 70 duplicate records, 121 unique records underwent title and abstract screening, of which 77 were excluded. Forty-four reports were sought for retrieval; 6 could not be retrieved, leaving 38 full-text reports assessed for eligibility. Thirteen reports were excluded at full-text review: different application of the magnetic field (*n* = 6), no assessment of osteogenic differentiation (*n* = 2), different nanoparticle composition (*n* = 2), lack of experimental data (*n* = 2), and an irrelevant cell type (*n* = 1). Twenty-five studies were included in the qualitative synthesis and 22 in the quantitative synthesis (Figure 1). Three studies had initially been summarized narratively only; their eligibility for pooling was reassessed during revision (Table 2). Cao et al. [34], Wong et al. [15], and Zhao et al. [35] remain in the narrative synthesis because they provide no in vitro ‘MNP + MF’ versus ‘MNP without MF’ contrast for any of the nine pre-specified outcomes.

### 3.2. Characteristics of Included Studies

The main characteristics of the 25 included studies are summarized in Table 3a (16 studies using human MSCs) and Table 3b (9 studies using animal MSCs), each arranged chronologically. The publications cover the period from 2010 to 2025. After verification against the source publications, the studies investigated MSCs derived from bone marrow (15 studies), adipose tissue (5 studies), Wharton’s jelly (1 study), umbilical cord (1 study), and dental pulp (1 study); in 2 studies the anatomical source of a commercial human MSC line was not reported. Animal MSCs were obtained from rats (*n* = 6) and mice (*n* = 3). Working MNP concentrations are reported in the tables alongside the physicochemical descriptors. Magnetic flux density ranged from 0.5 mT to approximately 1 T across static, low-frequency alternating, pulsed electromagnetic, and radiofrequency alternating regimens.

### 3.3. Risk of Bias

The results of risk of bias assessment by OHAT domains are shown in Figure 2 and Figure 3. The highest risk of bias was observed for the ‘personnel blinding’ domain. Information on its implementation was absent in all 25 included studies. Randomization of sample/culture vessel allocation was not explicitly reported in any study, although most described standard uniform cell seeding procedures; in line with the pre-specified adapted OHAT criteria, the absence of explicit reporting required classifying this domain as ‘probably high risk of bias’ across all included studies.

Outcome assessment blinding was not reported in most studies (probably high risk); low risk was assigned only to three studies in which histological sections from in vivo experiments were evaluated by independent blinded experts [18,40,48]. At the same time, all studies met the low risk criteria for data completeness and selective reporting: results were presented for all declared groups and time points without evidence of selective presentation. Cytotoxicity control of MNPs was performed at an acceptable level in most studies (standard MTT, CCK-8, WST-8, Alamar Blue, LDH assays, etc.); probably high or high risk was noted where direct viability assessment was not reported [14,15,37,48]. Independent replicates were explicitly stated in approximately two-thirds of studies. In the remaining studies, replicates could only be inferred indirectly from statistical procedures (probably high risk). Overall, no study exhibited low risk across all domains, which is characteristic of experimental studies.

### 3.4. Quantitative Synthesis (Meta-Analysis)

Biological, experimental, and methodological heterogeneity was substantial across all assessed outcomes. For five of the nine outcomes, I^2^ exceeded 75%. Because each outcome pools physically distinct magnetic-field regimes, the summary estimates in Table 4 and Figure 4 are exploratory composite models; primary interpretation relies on the stratified subgroup analyses, narrative synthesis, and the additional sensitivity analyses (Section 3.8).

#### 3.4.1. Alkaline Phosphatase Enzyme Activity

The pooled estimate from 14 studies was SMD = +4.42 (95% CI 0.97–7.86; *p* = 0.016), which is statistically significant at the uncorrected α level but does not survive Holm correction or trim-and-fill adjustment (Section 3.7). Crucially, the 95% prediction interval [−6.81; +15.64] broadly crossed zero, reflecting substantial uncertainty regarding the effect in any new experimental setting. The pooled direction remained positive across leave-one-out analyses, although the effect magnitude varied substantially (+2.74 to +4.93), and heterogeneity was critical (I2 = 84%; Figure 5). From the perspective of biological significance, the SMD magnitude indicates substantial enhancement of enzymatic activity in vitro, but the translatability of this effect to the clinical context remains hypothetical.

#### 3.4.2. Matrix Mineralization

Across 7 studies, SMD = +2.41 (0.03–4.80; *p* = 0.048; *I*^2^ = 66%): the only outcome with *I*^2^ < 75%, although statistical significance did not survive Holm correction. The direction of effect was consistent with other markers (Figure 6).

#### 3.4.3. Osteogenic Expression Markers

After separating the pools by analyte type, none of the molecular outcomes reached statistical significance at the uncorrected *p* level. ALP mRNA expression (now including Schäfer et al., 2010 [36]; *k* = 8) yielded SMD = +2.87 (−0.30–+6.04; *p* = 0.070; *I*^2^ = 80%). For osteocalcin mRNA (*k* = 4; SMD = +2.90; −3.18–8.98; *I*^2^ = 85%) and osteocalcin protein (*k* = 3; SMD = +4.47; −1.05–10.00; *I*^2^ = 52%), COL1 mRNA (*k* = 7; SMD = +1.73; −0.31–3.77; *I*^2^ = 74%), COL1 protein (*k* = 2; SMD = +4.94; −15.37–25.24; *I*^2^ = 34%), Runx2 mRNA (*k* = 8; SMD = +3.09; −2.07–8.24; *I*^2^ = 85%), and osteopontin mRNA (*k* = 5; SMD = +1.39; −2.22–5.00; *I*^2^ = 79%), confidence intervals crossed zero. Directionality remained predominantly positive. The corresponding forest plots are shown in Figure 7 (COL1 mRNA), Figure 8 (osteocalcin mRNA), Figure 9 (osteocalcin protein), and Figure 10 (osteopontin mRNA).

### 3.5. Subgroup Analysis

According to the Cochrane Handbook, subgroup analysis and meta-regression are methodologically justified only when at least 10 studies are included (*k* ≥ 10) to avoid insufficient statistical power and false-positive conclusions. Accordingly, subgroup analyses (by MF type, MSC source, and magnetic flux density level) and univariate meta-regression were performed only for ALP activity (*k* = 14). A subgroup contrast for COL1 mRNA under static fields is reported separately as an exploratory, non-pre-specified analysis because it was the least heterogeneous COL1 subgroup; it does not meet the *k* ≥ 10 criterion and should not be interpreted as a confirmed effect.

For subgroup analysis and meta-regression, magnetic field regimens were categorized into three pre-specified operational classes: (1) static magnetic fields (SMF; constant non-time-varying fields, *k* = 6); (2) alternating/time-varying continuous magnetic fields (LF-MF/SEMF; low-frequency sinusoidal or oscillating fields of 0.5–50 Hz; no radiofrequency field contributed to the ALP pool, *k* = 5); and (3) pulsed electromagnetic fields (PEMF; pulsed waveforms with defined pulse frequencies, *k* = 3). These classes are physically non-equivalent; the overall ALP pooled estimate is therefore an exploratory composite model, and primary interpretation is directed to Table 5. Subgroup analysis for ALP activity (Table 5) showed that the largest point estimate was observed with alternating/low-frequency fields (SMD = +10.76; 0.62–20.91; *k* = 5) compared to static (SMD = +1.98; not significant; *k* = 6) and pulsed fields (SMD = +1.18; not significant; *k* = 3). By cell source, a significant effect was maintained for bone marrow MSCs (SMD = +2.91; 0.18–5.64; *k* = 9), whereas the adipose-tissue subgroup was small and non-significant (SMD = +0.79; −6.03–7.60; *k* = 3; *I*^2^ = 82%), and the ‘other’ source subgroup comprised only two studies (*k* = 2). By magnetic flux density, effects were SMD = +3.54 (*k* = 5) below 50 mT and SMD = +5.48 (*k* = 9) at ≥50 mT. For COL1 mRNA, an exploratory static-field subgroup yielded SMD = +2.49 (0.25–4.73; *k* = 5; *I*^2^ = 60%). It should be emphasized that subgroups were small, and differences between them may reflect concomitant variations in protocols with cell type, matrix, and measurement methodology rather than a causal field-type effect.

### 3.6. Meta-Regression

To identify which study characteristics were associated with variation in ALP activity effect sizes, meta-regression was performed. For each study, the SMD and protocol characteristics (MF type, MSC source, etc.) were known, enabling assessment of whether these characteristics predict the effect magnitude (Table 6). Each characteristic (moderator) was analyzed separately in a univariate random-effects model with between-study variance estimated by REML. Moderators were coded as binary indicators (0/1), e.g., 1—study with alternating field, 0—without. Static field served as the reference category. For magnetic flux density, log_10_(*β* + 1) was used due to the wide range of values across studies (from 0.5 to 1 T). Alternating/time-varying continuous MF was coded as an indicator variable (1 vs. 0; static field served as the reference). The only association significant at the uncorrected *p* level was with alternating/time-varying MF: *β* = +7.87 (SE 2.87; *p* = 0.018). Thus, the observed SMD was on average approximately 8 Hedges’ g units higher in studies utilizing alternating or low-frequency fields. No associations were detected for other moderators (*p* > 0.30). Given the small number of studies (*k* = 14) and multiple tested moderators, these findings represent hypothesis-generating signals rather than definitive evidence of parameter optimization.

### 3.7. Sensitivity Analysis and Publication Bias

Sequential exclusion of individual studies (leave-one-out) confirmed that the positive direction of the pooled effect was preserved for ALP enzyme activity and COL1 mRNA; however, the magnitude of effect varied substantially (for ALP, SMD ranged from +2.74 to +4.93, decreasing to +2.74 upon omission of Russo et al. [17]). Egger’s test revealed evidence of significant funnel–plot asymmetry (positive regression intercept +4.09; *p* < 0.001; Figure 11), which may reflect small-study effects, between-study heterogeneity, and/or selective reporting rather than publication bias alone. Exploratory trim-and-fill analysis imputed 3 hypothetical studies on the negative side of the distribution; the adjusted estimate decreased from SMD = +4.42 to +1.34 (95% CI −1.60 to +4.27; *p* = 0.343), with the confidence interval encompassing zero. These exploratory sensitivity analyses suggest that the initial unadjusted pooled estimate may be substantially overestimated.

Holm correction for nine primary outcomes (Table 7) left no statistically significant effects at the family-wise α = 0.05: the smallest corrected p (Bonferroni) for ALP activity was 0.144. Thus, statistical significance of individual outcomes before correction should not be interpreted as confirmed effects; only the concordant positive direction of the majority of estimates appears more robust.

### 3.8. Additional Sensitivity Analyses

#### 3.8.1. Outlier Truncation

To assess the influence of anomalously large effect sizes arising from near-zero within-group standard deviations, all comparisons with |SMD| > 10 were excluded. For ALP activity, this removed 3 of 14 comparisons [12,17,43] and reduced the pooled estimate from SMD = +4.42 to SMD = +2.05, 95% CI +0.31 to +3.80, *p* = 0.025; heterogeneity decreased from I^2^ = 84% to 73%. For matrix mineralization, exclusion of Estévez et al., 2025 [43] gave SMD = +2.04, 95% CI +0.18 to +3.91, *p* = 0.037. ALP mRNA (SMD = +1.73, 95% CI −0.46 to +3.92, *p* = 0.101), Runx2 mRNA (SMD = +1.46, 95% CI −1.92 to +4.85, *p* = 0.331) and osteocalcin protein (SMD = +4.19, 95% CI −7.21 to +15.58, *p* = 0.134) remained non-significant after truncation, and osteocalcin mRNA, COL1 mRNA, COL1 protein and osteopontin mRNA contained no comparisons exceeding the threshold. Thus, the positive direction for ALP activity and mineralization did not depend on the most extreme comparisons, although their magnitude was roughly halved.

#### 3.8.2. Imputed Sample Size

Excluding the studies in which *n* = 3 was imputed (for ALP activity: [38,42,43,44,45]) yielded SMD = +5.63, 95% CI +0.15 to +11.12, *p* = 0.045 for ALP activity (*k* = 9) and SMD = +1.67, 95% CI +0.11 to +3.23, *p* = 0.041 for mineralization (*k* = 5), consistent with the primary analysis. For COL1 mRNA, omission of the single imputed study [42] shifted the estimate to SMD = +2.12, 95% CI +0.40 to +3.84, *p* = 0.025 (*k* = 6); all other outcomes remained non-significant. The imputation assumption therefore did not alter the direction of any pooled effect or the overall conclusions.

#### 3.8.3. Log Response Ratio

Re-expressing effects as lnRR, which does not divide by compressed standard deviations, gave lnRR = +0.415, 95% CI +0.151 to +0.678, *p* = 0.005 for ALP activity, lnRR = +0.307, 95% CI +0.075 to +0.539, *p* = 0.017 for ALP mRNA and lnRR = +0.262, 95% CI +0.119 to +0.406, *p* = 0.004 for mineralization. These correspond to approximately 51%, 36% and 30% higher mean values in the ‘MNP + MF’ group, respectively. For the remaining outcomes (Runx2 mRNA, osteocalcin mRNA, osteocalcin protein, COL1 mRNA, COL1 protein and osteopontin mRNA), confidence intervals crossed zero. All effects remained positive in direction, but heterogeneity was even higher on the lnRR scale (*I*^2^ = 85–100%), because the small delta-method variances of tightly controlled assays inflate *I*^2^. These lnRR results were not adjusted for multiplicity and are interpreted as a robustness check of effect direction rather than as confirmatory evidence. Full results for all nine outcomes are provided in Supplementary Table S3.

## 4. Certainty of Evidence (GRADE)

The overall certainty of evidence for each primary outcome was assessed using an adapted GRADE framework (Table 8). Because standard GRADE was developed primarily for clinical decision-making, we applied a predefined adaptation for preclinical in vitro evidence. The starting certainty for controlled experimental studies was set at ‘high’ by analogy to randomized trials due to tight baseline experimental control, followed by structured downgrading for risk of bias, critical heterogeneity, imprecision (wide CIs, small sample sizes), and funnel–plot asymmetry (possible small-study effects or publication bias). Upgrading for large effect size was conservatively scrutinized: because extremely large SMDs in small in vitro cohorts often arise from variance compression and small sample artifacts rather than definitive certainty, and because significance was eliminated following multiplicity adjustment (Holm) and trim-and-fill correction, no upgrade for large effect was applied in the primary certainty rating. Consequently, certainty was rated as ‘Very low’ for eight outcomes and ‘Low’ for matrix mineralization (owing to lower heterogeneity, *I*^2^ = 66%).

Thus, the highest (albeit only ‘low’) certainty was obtained for matrix mineralization. For ALP enzyme activity, certainty was ‘very low’ due to critical heterogeneity, funnel plot asymmetry, and loss of statistical significance after multiplicity and trim-and-fill adjustments. The direction of effect for most outcomes was concordantly positive; however, a positive direction of SMD does not constitute evidence of a biologically significant or clinically applicable effect.

## 5. Discussion

To our knowledge, the present review represents the first quantitative synthesis and meta-analysis evaluating the effect of combined magnetic field exposure and iron oxide nanoparticles on the osteogenic differentiation of mesenchymal stem cells. The analysis demonstrated a predominantly positive direction of effect across all nine investigated markers of osteogenesis (SMD from +1.39 to +4.94), although after correction for multiple comparisons, statistical significance was not retained for any outcome. This discrepancy between the high nominal effect size and the lack of formal statistical confirmation after correction is the central finding requiring careful discussion. Because these summary estimates pool physically distinct field regimes, they should be read as exploratory composite models; the stratified subgroup estimates (Table 5) are the primary basis for interpretation.

### 5.1. Biological Mechanisms

The observed effects are consistent with the concept of magnetomechanical transduction. An external MF acts on internalized or matrix-immobilized MNPs, generating local forces (from single to hundreds of piconewtons) that are transmitted to mechanosensitive receptors and the cytoskeleton. Force loading of integrin-linked adhesions and the actin network can open mechanosensitive ion channels—including Piezo-type mechanosensitive ion channel component 1 (Piezo1) [49], transient receptor potential vanilloid 4 (TRPV4) [50], and TREK-1—promoting calcium influx [13], activation of MAPK cascades [17], and nuclear translocation of YAP/TAZ [15], which in turn upregulate Runx2 and downstream osteogenic genes. Importantly, extracellular forces generated by functionalized magnetic scaffolds (ligand-tethered MNPs transmitting tension to cell-surface receptors) should be distinguished from shear and rotational stresses arising from endocytosed intracellular MNPs under time-varying fields; these two force-delivery modes may recruit overlapping but non-identical mechanotransduction pathways and likely contribute to between-study heterogeneity.

### 5.2. Exposure Characteristics and Sources of Heterogeneity

Meta-regression and subgroup analysis provide exploratory, hypothesis-generating signals suggesting that alternating/low-frequency and time-varying magnetic fields (SMD = +10.76; *k* = 5) may be associated with larger observed effect sizes than static fields in this dataset. This coefficient (*β* = +7.87, *p* = 0.018) must not be read as evidence that alternating fields are intrinsically superior: five moderators were tested at *k* = 14, confounding by nanoparticle formulation, scaffold mechanics, and assay timing remains unresolved, and the finding is explicitly exploratory. Magnetic flux density across the included studies ranged from 0.5 mT to approximately 1 T, and field magnitude alone is a poor predictor of the force acting on a cell. The translational force on a particle scales with the field gradient (*∇B*) and the particle’s magnetic moment rather than with B itself, so a strong but homogeneous field can exert little force, whereas a moderate field with a steep gradient can exert a large one, yet the gradient was rarely reported (e.g., 3.3–11.0 T/m in Hu et al. [37]). Waveform frequency and duty cycle determine whether particles are pulled, oscillated, or rotated; the mode of MNP localization (scaffold-tethered, membrane-bound, or internalized) determines where the force is applied; and high-frequency AMF adds a thermal component. Exposure duration (minutes per day to continuous exposure for 21 days), osteogenic supplementation of the medium, and assay time point varied independently of field strength. Because these parameters covary across studies, pooling across the intensity range without stratification inflates heterogeneity and limits mechanistic inference.

### 5.3. MNP Concentration, Field Intensity, and Cytotoxicity

In the included studies, working concentrations ranged from 10 to 60 µg/mL for suspended particles and from 0.1 to 30 wt.% for particles embedded in scaffolds (Table 3a,b). Several studies varied either MNP content [20,40,41] or field strength [46] alone, and Wu et al. [18] titrated both, exposing BMSCs to 0–200 µg/mL Fe_3_O_4_ under 0–200 mT and selecting 50 µg/mL with 100 mT as the combination most compatible with cell growth. Viability was not directly assessed in four studies [14,15,37,48], and none modeled toxicity as a joint function of concentration and field strength. Stronger fields and gradients increase magnetophoretic accumulation of particles at the cell surface and therefore the local particle dose, while high-frequency AMF adds heating (43 °C in Cao et al. [34]). Because the extracted comparisons were restricted to the non-toxic working concentration declared by the authors, the pooled estimates do not describe the concentration–toxicity boundary; future studies should report viability across a factorial grid of concentration and field parameters alongside osteogenic endpoints.

### 5.4. Consistency with in Vivo Data

Five included studies incorporated an in vivo animal defect repair component [18,35,39,40,48]. These investigations consistently reported enhanced bone regeneration in animal models (rabbit femoral defects, rat and mouse calvarial or osteochondral defects), demonstrating increased bone volume fraction (BV/TV) [18,35,39,40], elevated bone mineral density (BMD) [18,39], and improved trabecular thickness (Tb.Th) [48] under combined ‘MNP + MF’ stimulation. While these in vivo findings support the biological plausibility and translational promise of the approach, they cannot substitute for clinical efficacy data in humans.

### 5.5. Translational Engineering Challenges

Even if in vitro magnetomechanical stimulation proves robust under standardized protocols, clinical deep-tissue bone repair faces steep attenuation of magnetic field and gradient with distance from the source, governed approximately by inverse-square (and steeper) decay for realistic magnet geometries. Generating piconewton-scale forces uniformly across large anatomical volumes is therefore substantially harder than in culture plates, where fields are comparatively homogeneous. Engineering solutions may require implantable or wearable magnet arrays, locally concentrated MNPs within scaffolds, and patient-specific field planning—issues that currently limit direct clinical translation of the laboratory signal synthesized here.

### 5.6. Certainty of Evidence

Considering risk of bias, critical heterogeneity (*I*^2^ ≥ 75% for five of nine outcomes), small sample sizes, funnel plot asymmetry, loss of significance after Holm multiple testing correction, and reduction in the pooled effect in trim-and-fill analysis, the overall certainty of evidence is low to very low. Furthermore, 95% prediction intervals broadly spanned zero across all outcomes, indicating that in an individual newly conducted experiment, the effect may vary from strongly positive to absent or negligible. Therefore, statistical significance before correction was not equated with biological or clinical significance; the findings demonstrate a consistent positive direction of effect across laboratory studies rather than confirmed clinical efficacy.

## 6. Limitations

The present review has several limitations that must be considered when interpreting its results. First, the included studies were characterized by considerable heterogeneity. They differed in species and MSC source, type, size, coating, and concentration of nanoparticles, matrix composition, and outcome measurement methodologies. A separate source of heterogeneity is the differing physical nature of magnetic exposure. Static fields, pulsed/alternating fields, low-frequency magnetomechanical stimulation, and AMF hyperthermia may trigger different mechanisms of cellular response. Therefore, the pooled estimate reflects not a single standardized protocol but a broad class of magnetically controlled exposures. Heterogeneity is only partially mitigated by the use of standardized mean differences and a random-effects model, and is manifested in consistently high I^2^ values and wide prediction intervals that cross zero for all evaluated markers.

Second, most primary studies were characterized by small sample sizes (often only 3 biological replicates) combined with anomalously high standardized effect sizes (SMD > 2–4). This phenomenon is typical of highly controlled in vitro laboratory experiments and substantially increases the risk of overestimating the true biological and translational significance of the exposure. For several studies that did not report sample size, *n* was conservatively imputed (*n* = 3); sensitivity analyses with and without these studies are reported in Section 3.8. Furthermore, some numerical data were extracted from graphs in ImageJ by axis calibration and measurement of data-point and error-bar positions, and in certain studies results were normalized relative to controls, which increases the uncertainty of the final estimates.

Third, for ALP activity, evidence of funnel–plot asymmetry was detected (Egger’s test *p* < 0.001), which may reflect small-study effects and/or selective reporting rather than classical publication bias alone; exploratory trim-and-fill adjustment suggested potential overestimation, reducing the pooled SMD from +4.42 to +1.34 with the 95% confidence interval crossing zero. Moreover, when simultaneously testing nine outcomes, none retained significance after Holm correction, indicating the risk of false-positive conclusions at the uncorrected significance level. The review was not registered in a public registry (the full protocol is provided as Supplementary File S1), and conventional database coverage was limited to PubMed and Dimensions.

Finally, the choice of the primary comparison (‘MNP + MF’ vs. ‘MNP without MF’), optimal for isolating the effect of the magnetic field, led to the exclusion from the quantitative synthesis of studies lacking the corresponding control group, in particular, studies of magnetic hyperthermia (such data are summarized descriptively). The predominance of studies with positive results and the absence of preregistration for most primary studies further limit the certainty of conclusions.

## 7. Conclusions

A systematic review and meta-analysis of 25 experimental studies (22 quantitatively synthesized) demonstrate a predominantly positive direction of effect for combined ‘MNP + MF’ exposure on key markers of MSC osteogenic differentiation in vitro. However, critical heterogeneity (*I*^2^ > 75% for five of nine outcomes), loss of statistical significance after multiplicity correction, funnel–plot asymmetry for ALP activity, and prediction intervals that cross zero preclude claims of confirmed efficacy or of a single optimal magnetic regimen. Current evidence should be regarded as a low-certainty, hypothesis-generating signal for bone tissue engineering research.

To advance from preliminary experimental observations to reliable bone tissue engineering applications, future research must adopt standardized magnetic exposure protocols, transparently report sample sizes and technical replicates, implement investigator blinding and allocation concealment, and evaluate functional regeneration in validated critical-size defect models.

## Figures and Tables

**Figure 1:**
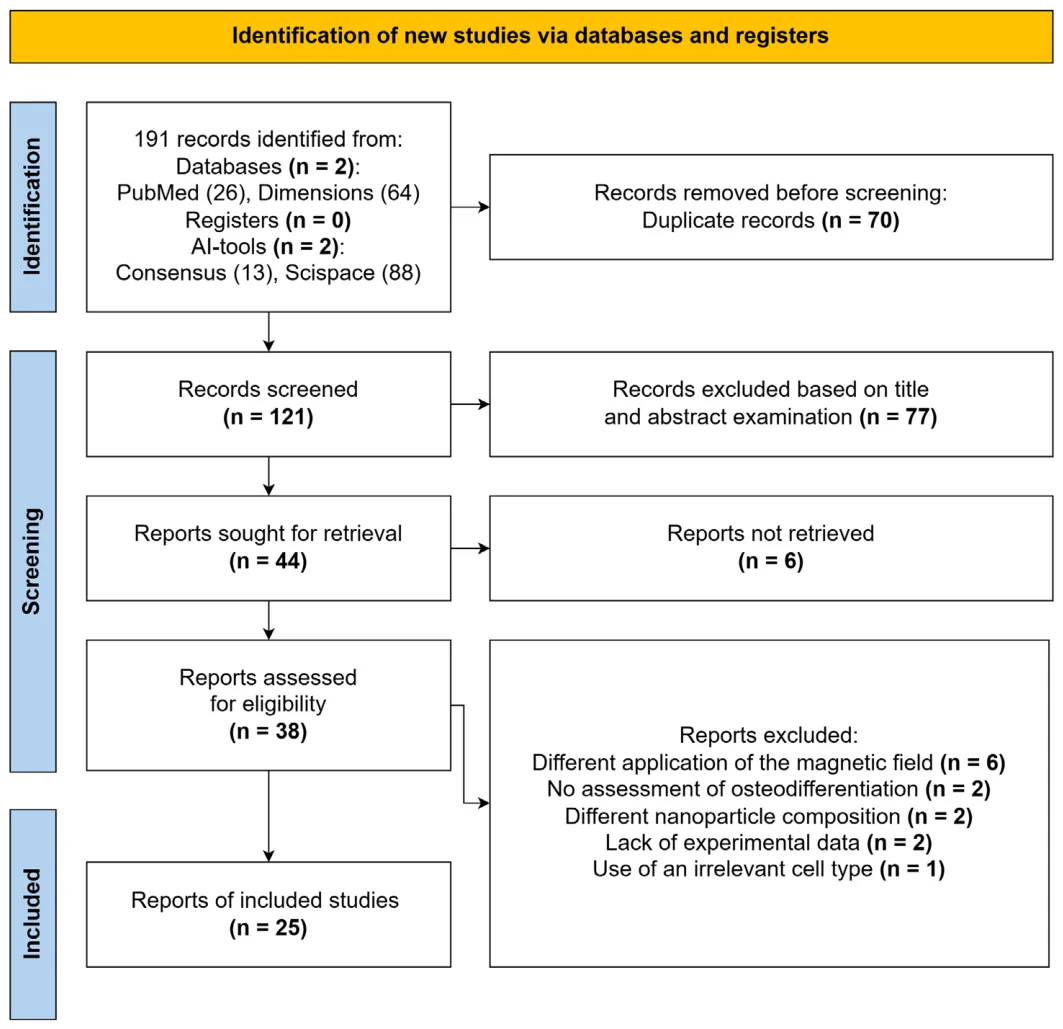
PRISMA flow diagram of study selection.

**Figure 2:**
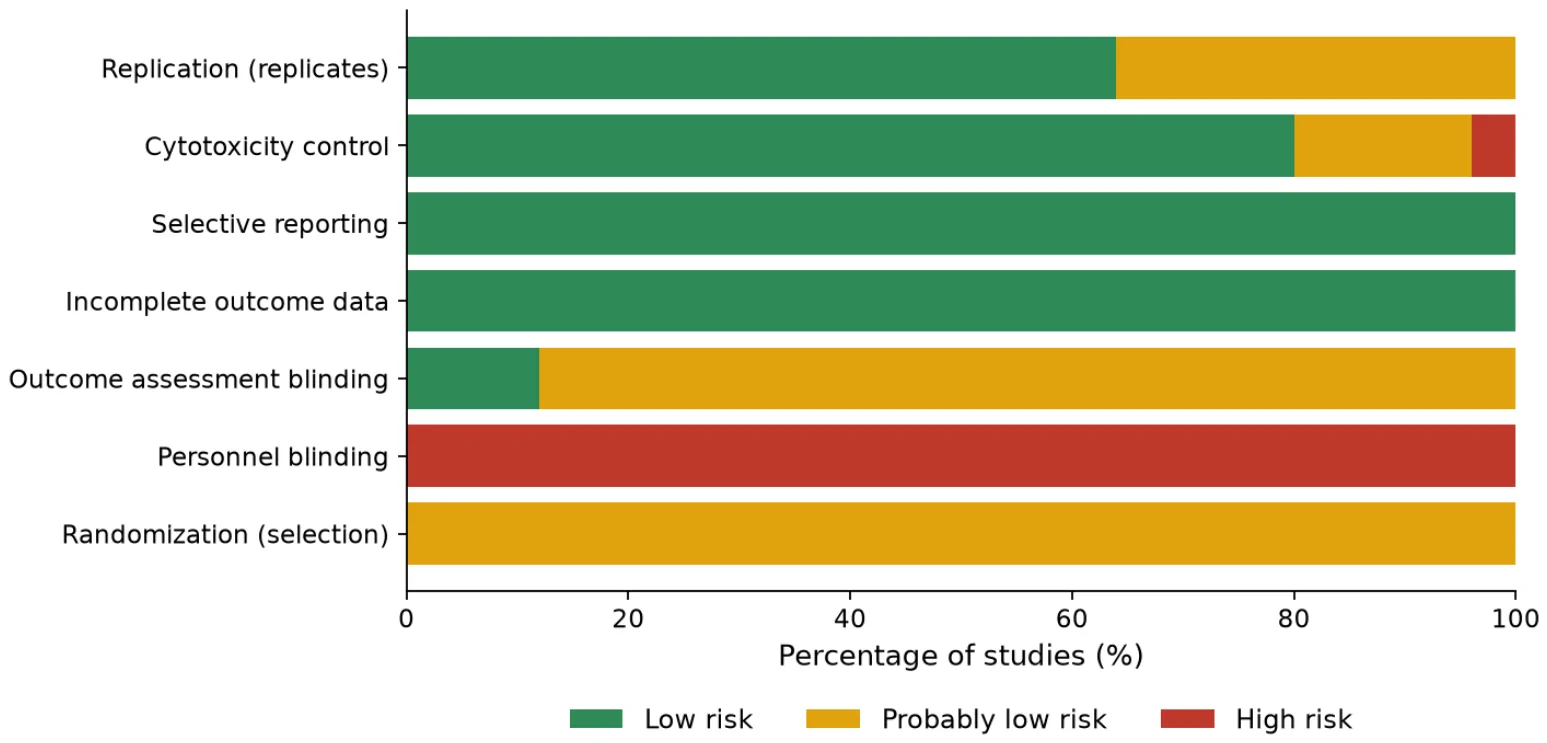
Summary risk of bias assessment by domain (percentage of studies).

**Figure 3:**
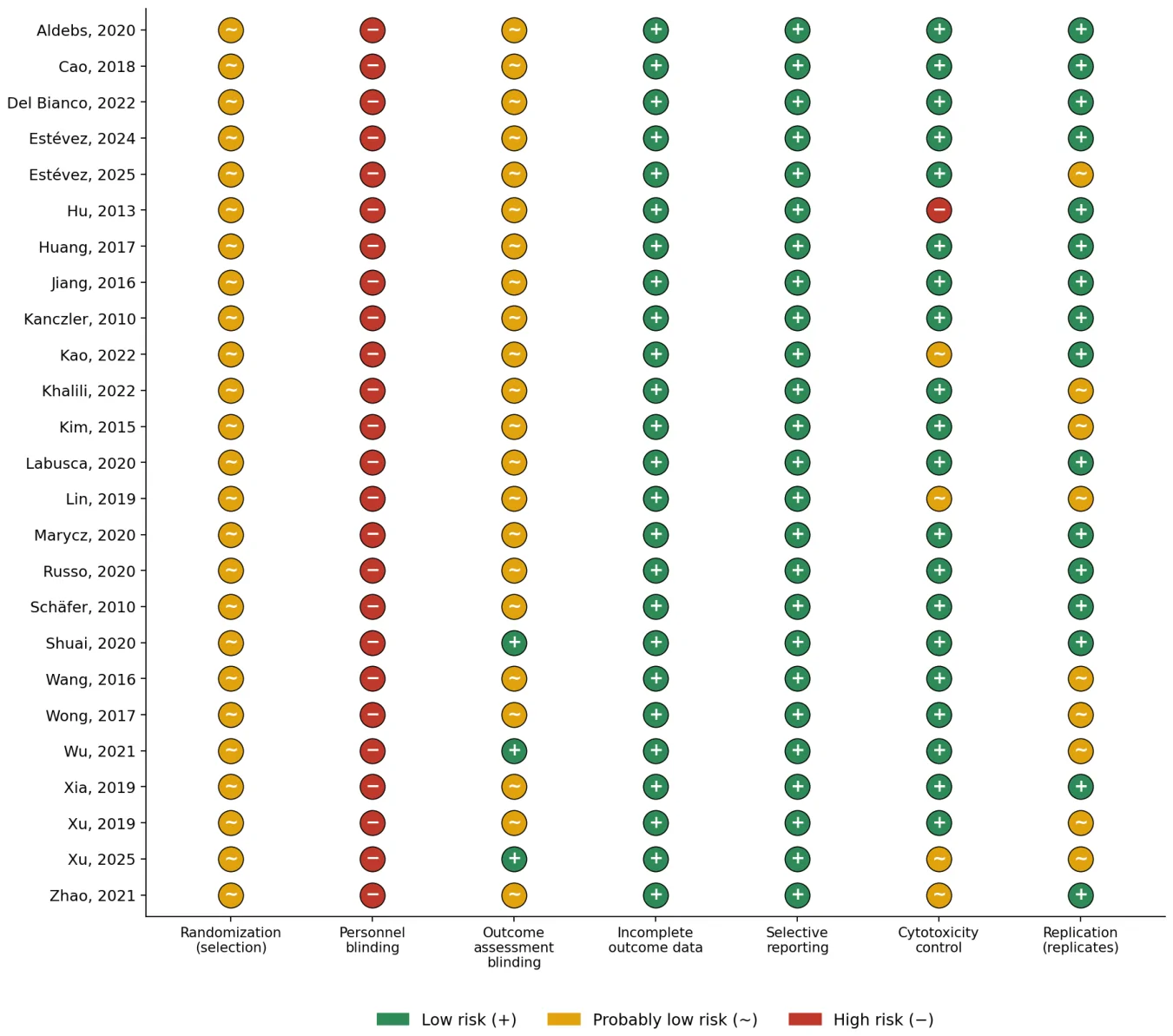
Risk of bias assessment (OHAT) for each study [11,12,13,14,15,16,17,18,19,20,34,35,36,37,38,39,40,41,42,43,44,45,46,47].

**Figure 4:**
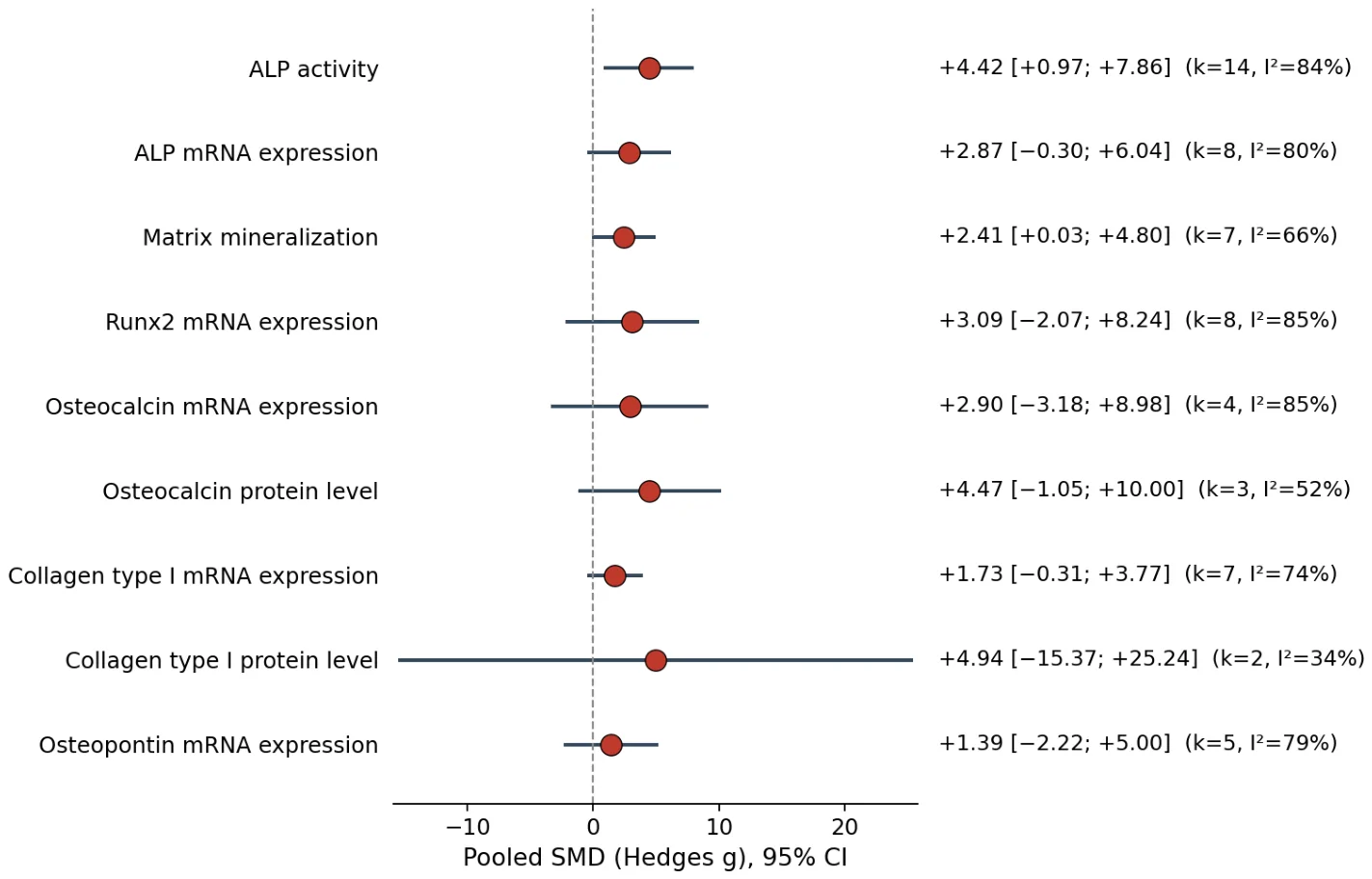
Summary pooled effect estimates across osteogenesis outcomes.

**Figure 5:**
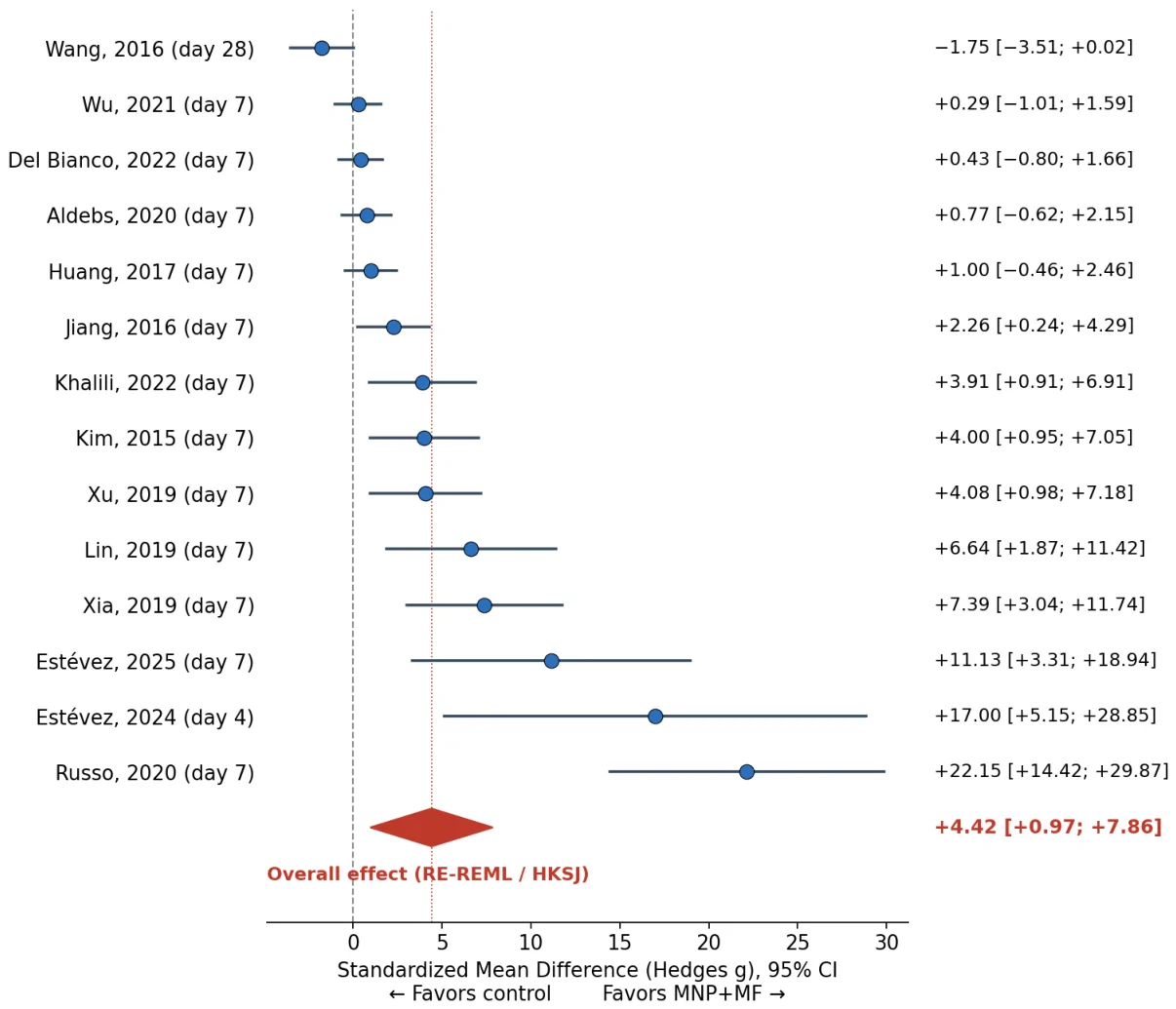
Forest plot: ALP activity. SMD (Hedges’ *g*) with 95% CI [11,12,14,16,17,18,38,39,41,42,43,44,45,46].

**Figure 6:**
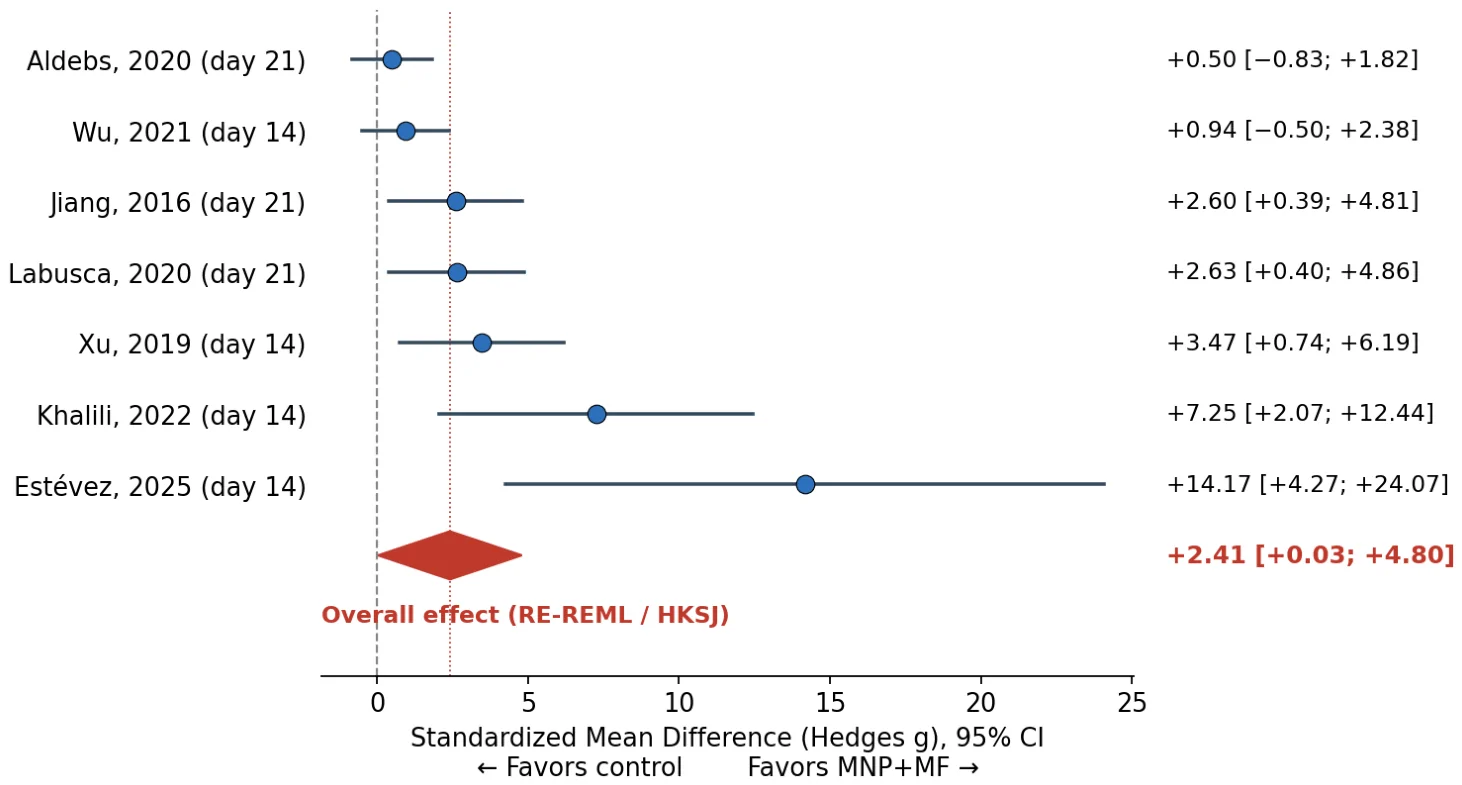
Forest plot: matrix mineralization. SMD (Hedges’ *g*) with 95% CI [11,16,18,19,42,43,46].

**Figure 7:**
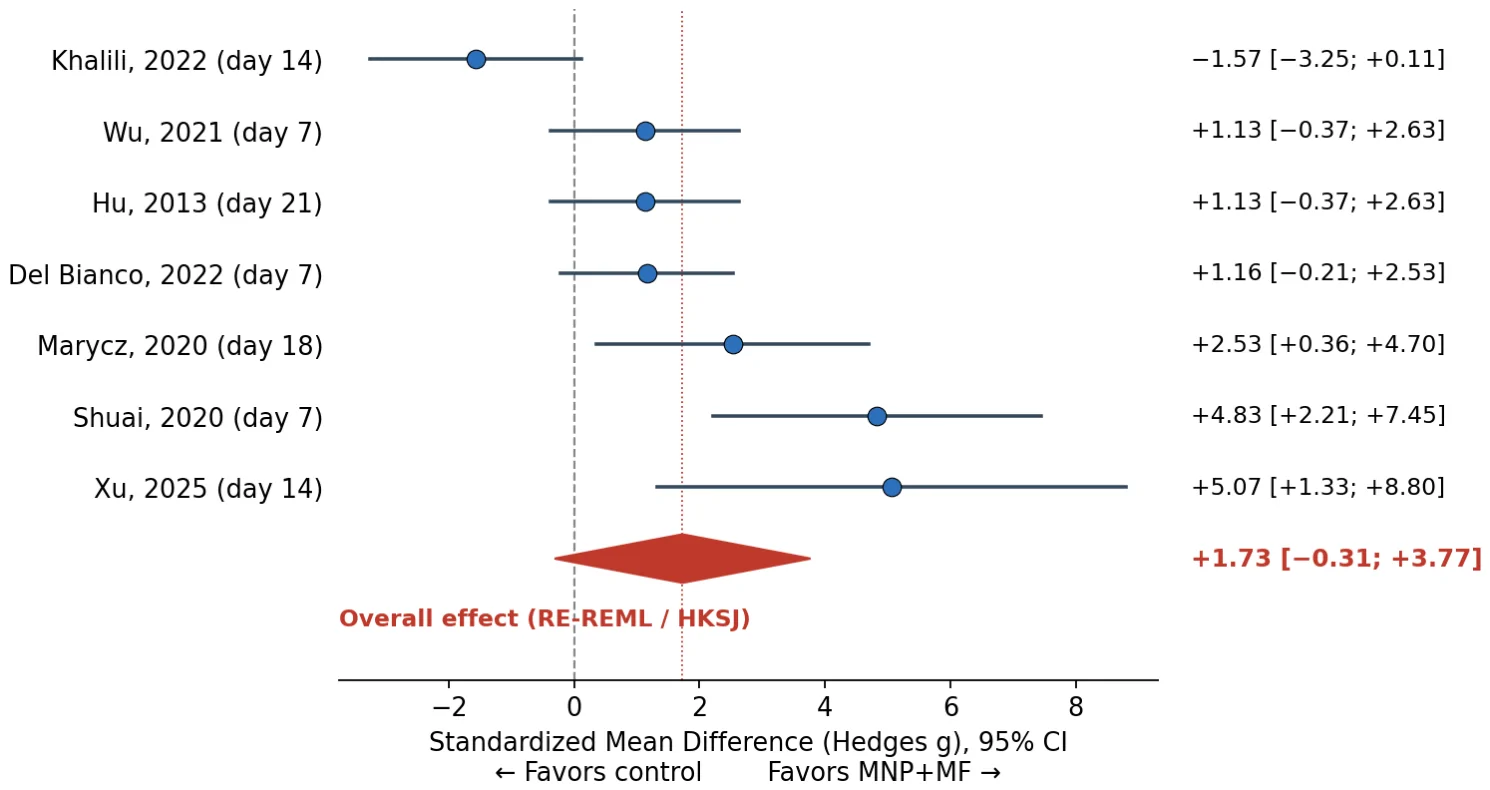
Forest plot: COL1 mRNA expression. SMD (Hedges’ *g*) with 95% CI [18,37,40,41,42,47,48].

**Figure 8:**
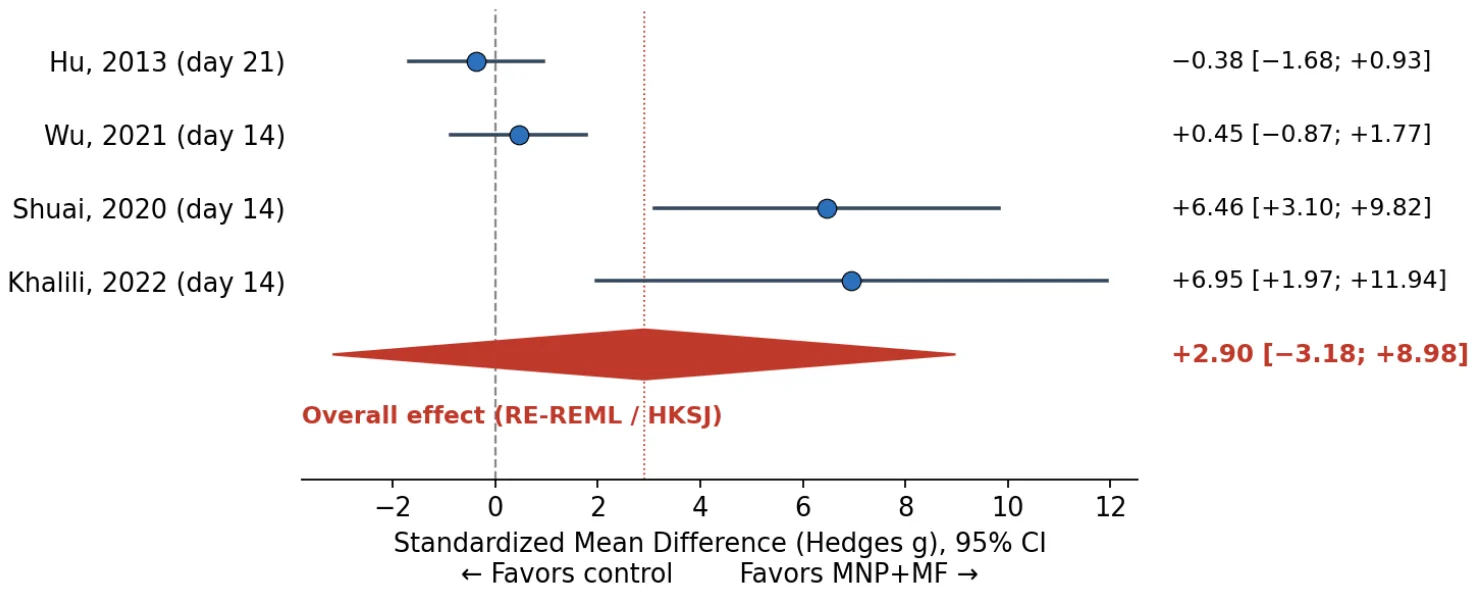
Forest plot: osteocalcin mRNA expression. SMD (Hedges’ *g*) with 95% CI [18,37,40,42].

**Figure 9:**
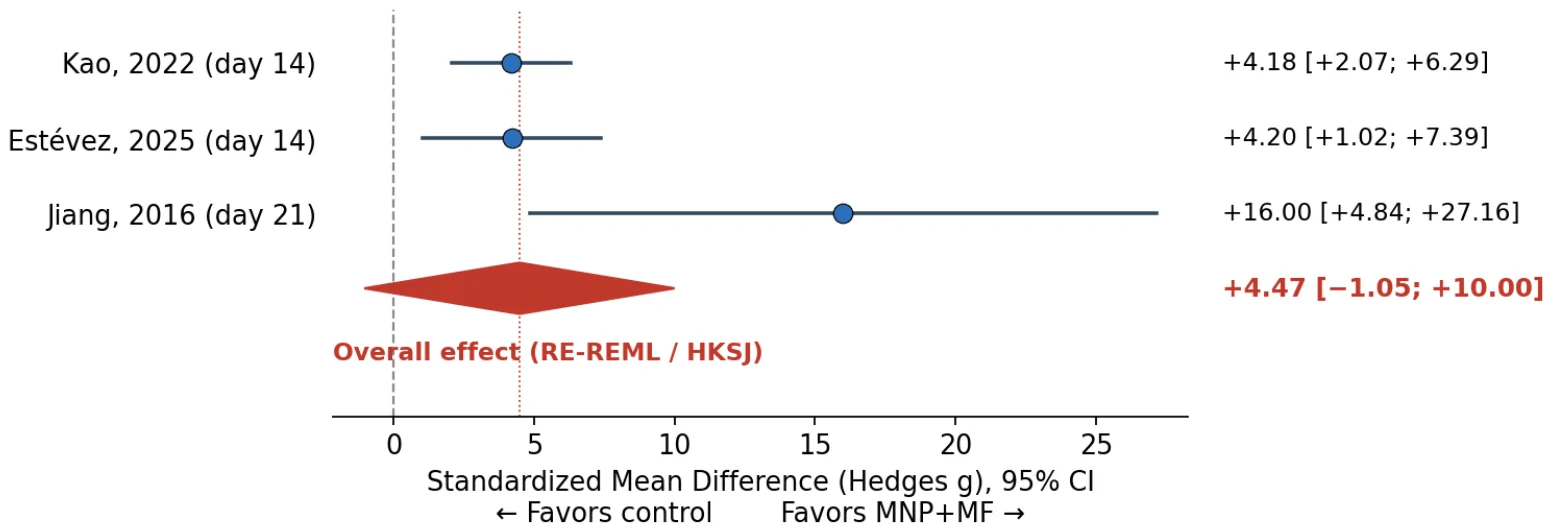
Forest plot: osteocalcin protein level. SMD (Hedges’ *g*) with 95% CI [16,20,43].

**Figure 10:**
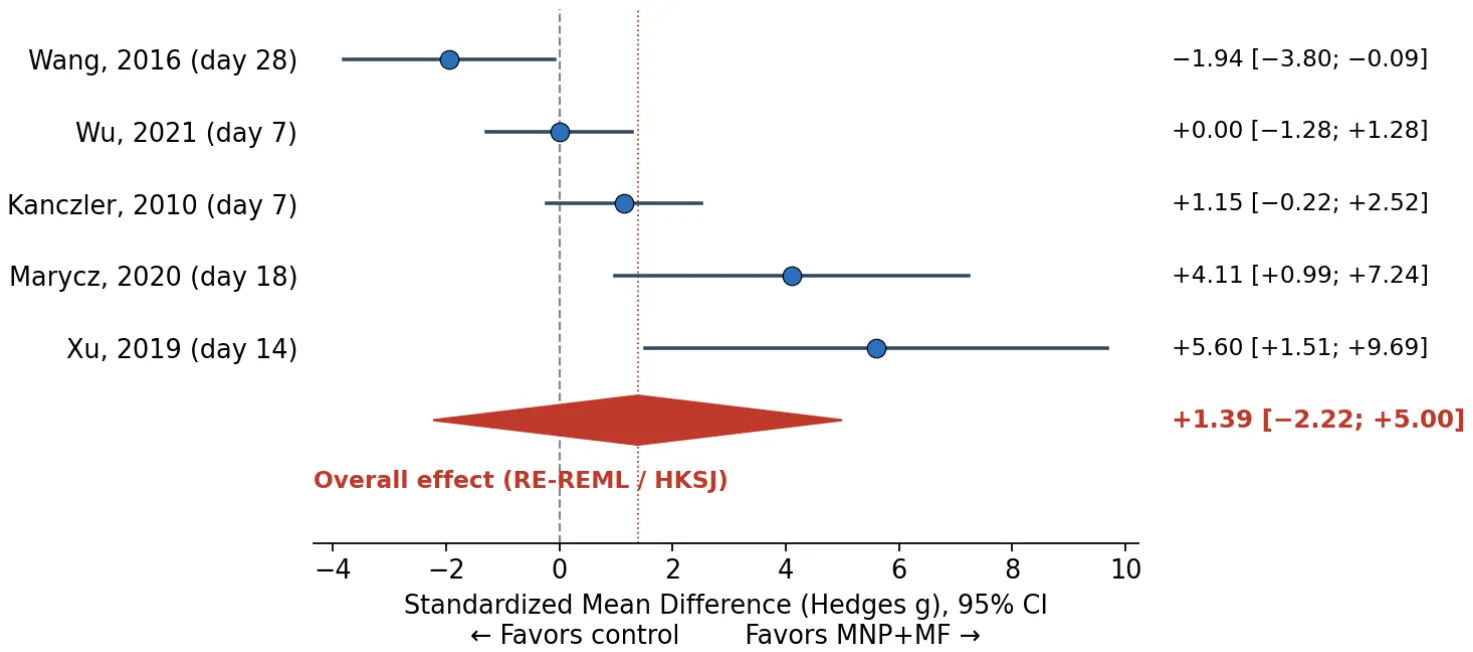
Forest plot: osteopontin mRNA expression. SMD (Hedges’ *g*) with 95% CI [13,18,44,46,47].

**Figure 11:**
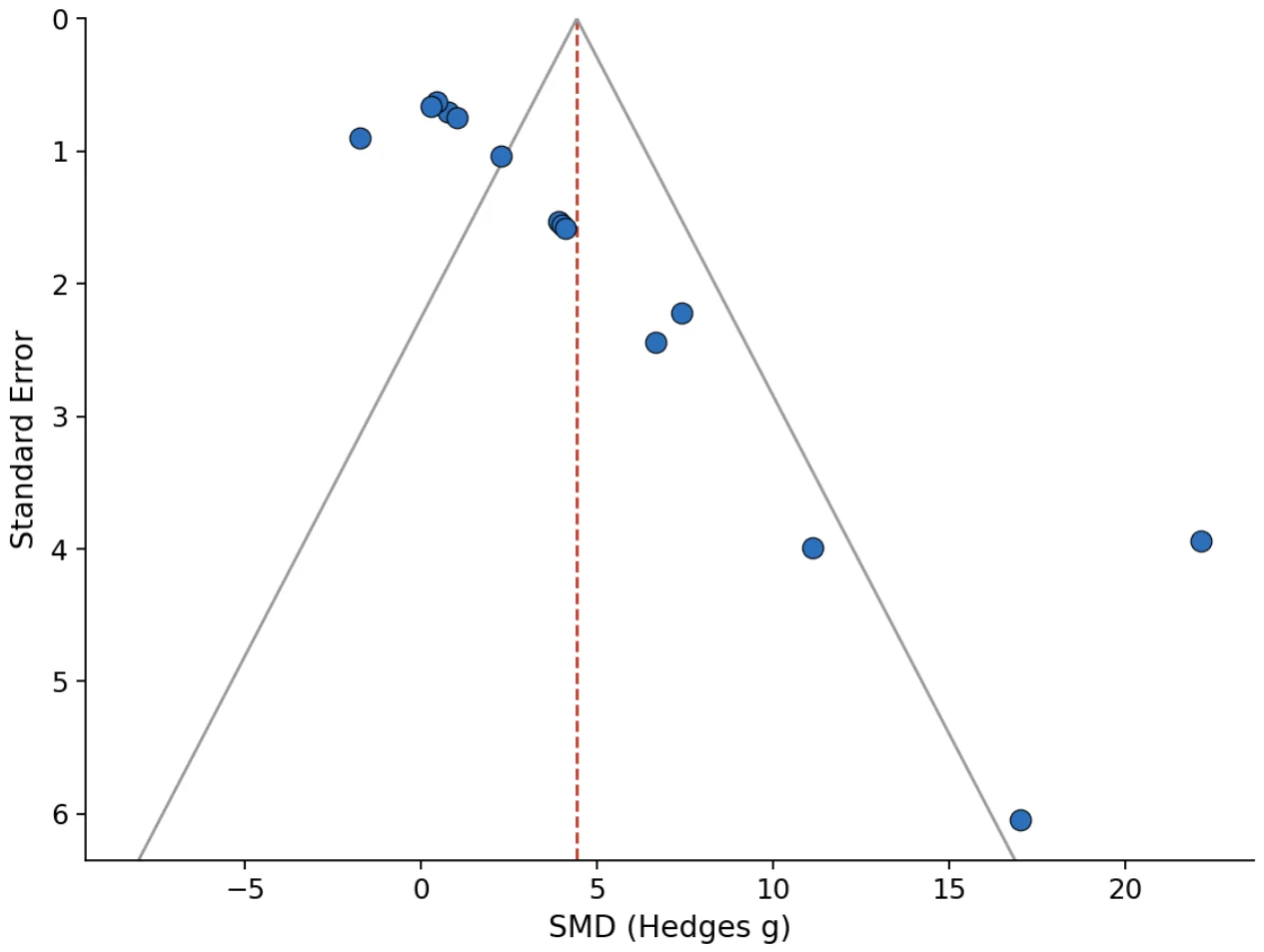
Funnel plot for ALP activity. Filled circles represent imputed studies (trim-and-fill method).

**Table 1:** Study inclusion and exclusion criteria (PICOS).

Category	Inclusion	Exclusion
P—Population	MSCs of any origin (bone marrow, adipose tissue, dental pulp, umbilical cord, Wharton’s jelly)	Studies on non-MSC cell types
I—Intervention	External magnetic field (static, alternating, or pulsed) applied to cells associated with iron oxide MNPs	MNPs only or MF only; non-magnetic nanoparticles; non-iron-oxide composition
C—Comparison	For qualitative synthesis: control group (without MNPs and/or without MF). For quantitative meta-analysis: control group exposed to MNPs without MF (‘MNP without MF’) to isolate the magnetic field effect.	No comparable control; for quantitative meta-analysis, absence of an ‘MNP without MF’ comparator group.
O—Outcomes	Osteogenesis markers: ALP, mineralization, Runx2, osteocalcin, collagen I, osteopontin (mRNA and/or protein)	No osteogenic differentiation outcomes
S—Study design	Controlled experimental studies (in vitro and/or in vivo)	Reviews, case reports, conference abstracts without data; non-English/non-Russian full texts
Additional	Extractable quantitative data (Mean ± SD or data obtainable by digital extraction)	Descriptive data only, no quantitative outcomes

**Table 2:** Eligibility for quantitative synthesis of the four studies initially summarized narratively.

Study	Available Groups and Outcomes	Assessment and Decision
Cao et al., 2018 [34]	Hydrogel with Fe_3_O_4_ under AMF (282 kHz, local heating to 43 °C) vs. plain hydrogel at 37 °C or water-bath heating to 43 °C; ALP activity	No ‘MNP without MF’ arm: every Fe_3_O_4_-hydrogel group was AMF-exposed, and the stimulus is thermal. Not pooled; narrative only.
Wong et al., 2017 [15]	MNP–RGD substrates with vs. without 86 mT SMF; ALP-positive cell area, nuclear RUNX2 and YAP localization (n not reported)	Image-based morphometric outcomes that do not correspond to any of the nine pre-specified outcomes. Not pooled; narrative only.
Zhao et al., 2021 [35]	In vitro: control, MF alone, PLGA + MF, and SPION-PLGA microspheres + MF (50 mT); in vivo: SPION-PLGA ± MF (300 mT); ALP, mineralization, BMD, BV/TV	No in vitro ‘MNP without MF’ arm; the only MNP ± MF contrast is in vivo (bone volume), outside the pre-specified in vitro outcomes. Not pooled; narrative only.

**Table 3:** (**a**) Key characteristics of included studies using human MSCs (*n* = 16), in chronological order. (**b**) Key characteristics of included studies using animal MSCs (*n* = 9), in chronological order.

(**a**)						
**Study**	**Species/MSC Source**	**MNP/Coating; Working Concentration**	**Size, nm**	**MF Type**	**Magnetic Flux Density (B)/Frequency**	**Outcomes Studied**
Kanczler et al., 2010 [13]	Human/BM	SPION/no coat; conc. 5–15 particles/cell	~10	Pulsed dyn.	n.r. (1–100 pN/particle); 1 Hz	mRNA: ALP, RUNX2, OCN, COL1
Schäfer et al., 2010 [36]	Human/BM	Fe_3_O_4_/dextran; PVP; conc. 60 µg/mL	60; 30	SMF	600 mT	ALP; min.
Hu et al., 2013 [37]	Human/BM	SPION/RGD, PAMAM; conc. 25 µg/well	250 ^1^	Pulsed dyn.	60–120 mT; 1 Hz	mRNA: COL1, RUNX2, OCN, OPN
Kim et al., 2015 [38]	Human/BM	Fe_3_O_4_/no coat; conc. 50 µg/mL	50	SEMF	1 mT; 45 Hz	ALP; mRNA: ALP, RUNX2, COL1, OPN
Wong et al., 2017 [15]	Human/n.r.	Fe_3_O_4_/PEG-RGD; conc. 15–18 particles/µm^2^	10	SMF, grad.	86 mT	mRNA: RUNX2, OPN; prot.: YAP
Xia et al., 2019 [39]	Human/DP	Fe_3_O_4_/n-HA, CPC; conc. 1.5 wt.% in scaffold	~20	SMF	35 mT ^5^	ALP; mRNA: RUNX2, OCN; min.; BMD, BV/TV in vivo
Aldebs et al., 2020 [11]	Human/AT	SPION/−COOH; conc. 0.5% *v*/*v* in hydrogel	10	PEMF	1 mT; 15 Hz	ALP; min.
Labusca et al., 2020 [19]	Human/AT	Fe_3_O_4_/oleic acid; conc. 20 µg/mL	10–12	LMF/HMF ^2^	LMF 0.5 mT, HMF 14.7 mT	ALP; mRNA: RUNX2, OCN, OPN; prot.: OCN
Russo et al., 2020 [17]	Human/n.r.	Fe_3_O_4_/PCL; conc. 20 wt.% in PCL composite	200 ^3^	Alternating	50 mT; 0.5 Hz	ALP
Shuai et al., 2020 [40]	Human/UC	Fe_3_O_4_/PLLA; conc. 0–8 wt.% in scaffold	30	SMF	55–65 mT ^5^	ALP; mRNA: ALP, RUNX2, COL1; prot.: OCN; min.; BV/TV in vivo
Wu et al., 2021 [18]	Human/BM	Fe_3_O_4_/PLGA; conc. 50 µg/mL	~7	SMF	100 mT	ALP; mRNA: ALP, RUNX2, COL1, OPN; prot.: COL1; min.
Del Bianco et al., 2022 [41]	Human/BM	SPION/silk fibroin; conc. 0.0625–0.625 mg/mL	~10	SMF	~160 mT	ALP; mRNA: ALP, COL1, RUNX2, SPARC
Kao et al., 2022 [20]	Human/WJ	Fe_3_O_4_/PCL, CS; conc. 0–5 wt.% in scaffold	~20	SMF	150 mT	ALP; mRNA: RUNX2, BMP2; min.
Khalili et al., 2022 [42]	Human/AT	SPION/PAMAM; conc. 0.1% *w*/*v* in PCL	7–8 ^1^	PEMF	30 mT; 75 Hz	ALP; mRNA: ALP, COL1; prot.: COL1; min.
Estévez et al., 2024 [12]	Human/BM	SPION/DMSA, RGD; conc. 20 µg Fe/mL	~12	LF-MF	250 mT; 1 Hz	ALP; mRNA: ALP, RUNX2
Estévez et al., 2025 [43]	Human/BM	SPION/PEI, Col; conc. 2.25–2.32 µg Fe/cm^2^ scaffold	~12	LF-MF	250 mT; 1 Hz	ALP; mRNA: ALP, RUNX2; min.
(**b**)						
**Study**	**Species/MSC Source**	**MNP/Coating; Working** **Concentration**	**Size, nm**	**MF Type**	**Magnetic Flux Density (B)/Frequency**	**Outcomes Studied**
Jiang et al., 2016 [16]	Rat/BM	Fe_3_O_4_/BSA; conc. 50 µg/mL	80	SMF	~1 T	ALP; mRNA: ALP, RUNX2, COL1, OCN, OPN; min.
Wang et al., 2016 [44]	Rat/AT	SPION/no coat; conc. 50 µg/mL	20–30	SMF	500 mT	ALP ^4^; min.
Huang et al., 2017 [45]	Rat/BM	Fe_3_O_4_/CPC; conc. 5 wt.% in CPC	20–30	PEMF	100 mT; n.r.	ALP; mRNA: ALP, RUNX2, OCN, BSP
Cao et al., 2018 [34]	Rat/BM	Fe_3_O_4_/chitosan, PEG; conc. 30 wt.% in hydrogel	40–50	AMF (hyperthermia)	282 kHz	ALP
Lin et al., 2019 [14]	Rat/BM	Fe_3_O_4_/mineral. Col; conc. 8.5 wt.% in coating	~200	Period. SMF	150–280 mT	ALP; mRNA: ALP; min.
Xu et al., 2019 [46]	Rat/BM	Fe_3_O_4_/no coat; conc. 10 µg/mL	10–15	SEMF	1–4 mT; 50 Hz	ALP; mRNA: ALP, RUNX2, OCN; prot.: RUNX2
Marycz et al., 2020 [47]	Mouse/AT	γ-Fe_2_O_3_/PU-PLLA; conc. 1–5 wt.% in sponge	~30	SMF	0.2 T	ALP; mRNA: COL1, RUNX2, OCN; prot.: OCN
Zhao et al., 2021 [35]	Mouse/BM	Fe_3_O_4_/PLGA; conc. 1.16–1.83 wt.% in microspheres	8–10	SMF	50 mT ^5^	ALP; mRNA: RUNX2, OCN; min.; BV/TV in vivo
Xu et al., 2025 [48]	Mouse/BM	Fe_3_O_4_@SiO_2_/gel; conc. 10% *w*/*v* prepolymer	~8 ^3^	SMF	15 mT	mRNA: RUNX2, COL1; in vivo Tb.Th

Notes (Table 3a,b). Species/MSC source: BM—bone marrow; AT—adipose tissue; WJ—Wharton’s jelly; DP—dental pulp; UC—umbilical cord; n.r.—not reported (for MSC source: anatomical source of a commercial line not reported). MNPs: SPION—superparamagnetic iron oxide nanoparticles; Fe_3_O_4_—magnetite; γ-Fe_2_O_3_—maghemite. Coatings: DMSA—dimercaptosuccinic acid; PEG—polyethylene glycol; PAMAM—polyamidoamine dendrimer; PVP—polyvinylpyrrolidone; PEI—polyethylenimine; BSA—bovine serum albumin; RGD—Arg-Gly-Asp peptide; PCL—polycaprolactone; CS—calcium silicate; PLGA—poly(lactic-co-glycolic acid); PLLA—poly-L-lactic acid; PU—polyurethane; CPC—calcium phosphate cement; n-HA—nanohydroxyapatite; Col—collagen; −COOH—carboxyl groups; no coat—uncoated. MF types: SMF—static magnetic field; PEMF—pulsed electromagnetic field; AMF—alternating magnetic field; SEMF—sinusoidal electromagnetic field; LMF/HMF—low-/high-frequency alternating field; LF-MF—low-frequency oscillating field; dyn.—dynamic field produced by moving magnet arrays; period.—periodic SMF on/off; grad.—gradient field. Outcomes: ALP—alkaline phosphatase activity; min.—extracellular matrix mineralization; mRNA—relative gene expression (RT-qPCR); prot.—protein content or nuclear localization; RUNX2—runt-related transcription factor 2; OCN—osteocalcin; OPN—osteopontin; COL1—type I collagen; BSP—bone sialoprotein; SPARC—osteonectin; BMP2—bone morphogenetic protein 2; YAP—Yes-associated protein; BMD—bone mineral density; BV/TV—bone volume fraction; Tb.Th—trabecular thickness. ^1^ Hydrodynamic diameter. ^2^ LMF: 0.5 mT, 7 Hz; HMF: 14.7–21.6 mT, 113 kHz. ^3^ Core size of Fe_3_O_4_ or Fe_3_O_4_@SiO_2_. ^4^ ALP-positive cell area. ^5^ In vitro value shown; in vivo exposure 40–70 mT (Shuai et al. [40]) and 300 mT (Xia et al. [39]; Zhao et al. [35]).

**Table 4:** Summary meta-analysis results (‘MNP + MF’ vs. ‘MNP without MF’). Outcomes statistically significant before multiple comparison correction are shown in bold.

Outcome	k	SMD (Hedges’ g)	95% CI	*p*	I^2^, %	PI
**ALP enzyme activity**	14	+4.42	+0.97; +7.86	0.016	83.62%	−6.81; +15.64
ALP mRNA expression	8	+2.87	−0.30; +6.04	0.070	80.50%	−5.20; +10.93
Matrix mineralization	7	+2.41	+0.03; +4.80	0.048	65.99%	−2.02; +6.85
Runx2 mRNA expression	8	+3.09	−2.07; +8.24	0.200	85.26%	−10.46; +16.63
Osteocalcin mRNA expression	4	+2.90	−3.18; +8.98	0.226	85.07%	−14.25; +20.05
Osteocalcin protein level	3	+4.47	−1.05; +10.00	0.073	52.38%	−11.84; +20.78
COL1 mRNA expression	7	+1.73	−0.31; +3.77	0.083	74.36%	−3.45; +6.91
COL1 protein level	2	+4.94	−15.37; +25.24	0.199	34.26%	n/c
Osteopontin mRNA expression	5	+1.39	−2.22; +5.00	0.346	79.18%	−7.60; +10.38

Notes. n/c—not calculable (k < 3): a 95% prediction interval requires at least three studies. COL1 protein (k = 2) is retained because it is one of the nine pre-specified outcomes; it was analyzed with the same Hedges’ g/REML/HKSJ model as the other outcomes, and its pooled estimate is regarded as descriptive only. PI—95% prediction interval.

**Table 5:** Subgroup analysis for alkaline phosphatase activity.

Subgroup (Outcome: ALP Enzyme Activity)	k	SMD	95% CI	I^2^, %
MF type: static	6	+1.98	−1.65; 5.62	80
MF type: alternating/low-frequency	5	+10.76	0.62; 20.91	84
MF type: pulsed (PEMF)	3	+1.18	−1.62; 3.98	44
MSC source: bone marrow	9	+2.91	0.18; 5.64	72
MSC source: adipose tissue	3	+0.79	−6.03; 7.60	82
MSC source: other (UC/DP/n.r.)	2	+14.41	−79.23; 108.05	91
Magnetic flux density: <50 mT	5	+3.54	0.55; 6.54	70

**Table 6:** Univariate meta-regression for ALP activity.

Moderator (Outcome: ALP Enzyme Activity)	*β*	SE	*p*
MF type: alternating/time-varying (0/1)	+7.87	2.87	0.018
MF type: pulsed (0/1)	−3.39	3.74	0.382
Source: bone marrow (0/1)	−1.19	3.43	0.736
Species: animal (0/1)	−3.52	3.25	0.300
log_10_(magnetic flux density, mT + 1)	+0.01	1.88	0.997

Notes. *τ*^2^ was estimated by REML and equaled 24.0 for all univariate models.

**Table 7:** Multiple comparison correction for primary outcomes (α = 0.05).

Outcome	*p* (Uncorrected)	*p* (Bonferroni)	*p* (Holm)	Significant After Holm
ALP enzyme activity	0.0160	0.1437	0.1437	No
ALP mRNA	0.0699	0.6287	0.4890	No
Mineralization	0.0479	0.4310	0.3831	No
Runx2 mRNA	0.1999	1.0000	0.7970	No
Osteocalcin mRNA	0.2263	1.0000	0.7970	No
Osteocalcin protein	0.0734	0.6608	0.4890	No
COL1 mRNA	0.0834	0.7508	0.4890	No
COL1 protein	0.1992	1.0000	0.7970	No
Osteopontin mRNA	0.3463	1.0000	0.7970	No

**Table 8:** GRADE certainty of evidence assessment for primary outcomes.

Outcome	k	SMD [95% CI]	Downgrading Factors	Upgrading Factors	Certainty
ALP enzyme activity	14	+4.42 [0.97; 7.86]	Risk of bias (−1); heterogeneity *I*^2^ = 84% (−2); funnel–plot asymmetry/possible small-study effects (−1)	None (large effect not upgraded due to instability after Holm/trim-and-fill)	Very low
Mineralization	7	+2.41 [0.03; 4.80]	Risk of bias (−1); heterogeneity *I*^2^ = 66% (−1); imprecision (−1)	None (large effect not upgraded due to small sample size and Holm non-significance)	Low
Osteocalcin mRNA	4	+2.90 [−3.18; 8.98]	Risk of bias (−1); heterogeneity *I*^2^ = 85% (−2); imprecision (−1)	– (effect not confirmed)	Very low
Osteocalcin protein	3	+4.47 [−1.05; 10.00]	Risk of bias (−1); heterogeneity *I*^2^ = 52% (−1); imprecision (−1)	– (effect not confirmed)	Very low
COL1 mRNA	7	+1.73 [−0.31; 3.77]	Risk of bias (−1); heterogeneity *I*^2^ = 74% (−1); imprecision (−1)	– (effect not confirmed)	Very low
COL1 protein	2	+4.94 [−15.37; 25.24]	Risk of bias (−1); heterogeneity (−1); imprecision (−2)	– (effect not confirmed)	Very low
ALP mRNA	8	+2.87 [−0.30; 6.04]	Risk of bias (−1); heterogeneity *I*^2^ = 80% (−2); imprecision (−1)	– (effect not confirmed)	Very low
Runx2 mRNA	8	+3.09 [−2.07; 8.24]	Risk of bias (−1); heterogeneity *I*^2^ = 85% (−2); imprecision (−1)	– (effect not confirmed)	Very low
Osteopontin mRNA	5	+1.39 [−2.22; 5.00]	Risk of bias (−1); heterogeneity *I*^2^ = 79% (−1); imprecision (−1)	– (effect not confirmed)	Very low

Notes. Dose–response relationships were not systematically reported and were not used for upgrading. Upgrading for large effect was not applied because observed extreme SMDs in small in vitro samples were not robust to multiple comparison correction (Holm) and sensitivity adjustments.

## Data Availability

All data supporting the findings of this study are available in the Supplementary Materials. The analysis code and extracted dataset are available upon request from the corresponding author.

## Supplementary Materials

The following supporting information can be downloaded at: https://www.mdpi.com/article/10.3390/bioengineering13101143/s1, File S1: Study protocol; Table S1: Search queries; Table S2: Data extracted from included studies; Table S3: Additional sensitivity analyses (outlier truncation, imputed n, and lnRR).

## Abbreviations

The following abbreviations are used in this manuscript:

ADSC	adipose-derived stem cell
ALP	alkaline phosphatase
AMF	alternating magnetic field
BM	bone marrow
BMD	bone mineral density
BMP	bone morphogenetic protein
BMSC	bone marrow stromal cell
BV/TV	bone volume fraction
CI	confidence interval
COL1	type I collagen
DPSC	dental pulp stem cell
GRADE	Grading of Recommendations Assessment, Development and Evaluation
HKSJ	Hartung–Knapp–Sidik–Jonkman
LF-MF	low-frequency oscillating magnetic field
lnRR	natural logarithm of the response ratio
MAPK	mitogen-activated protein kinase
MeSH	Medical Subject Headings
MF	magnetic field
MNP	magnetic nanoparticle
MSC	mesenchymal stem cell
OCN	osteocalcin
OHAT	Office of Health Assessment and Translation
OPN	osteopontin
PEMF	pulsed electromagnetic field
PI	prediction interval
PICOS	Population, Intervention, Comparison, Outcomes, and Study design
Piezo1	Piezo-type mechanosensitive ion channel component 1
PRISMA	Preferred Reporting Items for Systematic Reviews and Meta-Analyses
REML	restricted maximum likelihood
RT-qPCR	reverse transcription quantitative polymerase chain reaction
Runx2	runt-related transcription factor 2
SEMF	sinusoidal electromagnetic field
SMD	standardized mean difference
SMF	static magnetic field
SPION	superparamagnetic iron oxide nanoparticle
Tb.Th	trabecular thickness
TREK-1	TWIK-related potassium channel-1
TRPV4	transient receptor potential vanilloid 4
YAP/TAZ	Yes-associated protein/transcriptional coactivator with PDZ-binding motif
