# Supplementary material for: Effect of Combined Iron Oxide Magnetic Nanoparticles and Magnetic Field Exposure on Osteogenic Differentiation of Mesenchymal Stem Cells: A Systematic Review and Meta-Analysis
Source: Bioengineering (Basel). 2026 Sep 30;13(10):1143. doi: 10.3390/bioengineering13101143 (PMC13701717; doi:10.3390/bioengineering13101143)
Supplement: Supplementary file 1 [file bioengineering-13-01143-s001.zip › File_S1_Study_protocol.pdf]

Effect of combined iron oxide magnetic nanoparticles and magnetic fields on osteogenic differentiation of mesenchymal stromal cells: a systematic review protocol

Systematic review and meta-analysis protocol | Retrospective methodological record

Authors: Maksim Solopov, Roman Ishchenko, Viktor Turchin, Viktoria Bushe, Anna Kavelina, Gulnara Akopian, Elizaveta Lebed and Andrey Popandopulo.

Affiliation: V.K. Gusak Institute of Emergency and Reconstructive Surgery, 283045 Donetsk, Russia.

Correspondence: m.solopov@iursdn.ru

Keywords: mesenchymal stromal cells; iron oxide nanoparticles; magnetic fields; osteogenic differentiation; bone tissue engineering; meta-analysis.

Protocol status: This record was prepared after the review analyses documented in the supplied manuscript. It is not evidence of prospective registration or that every method was specified before results were known. No verifiable registration identifier was supplied; the manuscript contains an unfilled OSF identifier. Original dated search logs and decisions should be deposited with any public release.

Abstract

Objective: To evaluate whether an external magnetic field changes osteogenic differentiation in mesenchymal stromal cells associated with iron oxide magnetic nanoparticles, compared with matched nanoparticle exposure without a field.

Introduction: Magnetically responsive cells and scaffolds are being studied for bone tissue engineering, but cell source, particle formulation, field regime, and outcome assay differ substantially among experiments. An explicit review method is needed to distinguish the effect of the field from effects of the nanoparticles.

Methods: Controlled experimental studies of human or animal mesenchymal stromal cells were eligible for qualitative synthesis when cells were associated with iron oxide nanoparticles and exposed to an external magnetic field. Quantitative synthesis required a nanoparticle-exposed comparator without a field and extractable group data. PubMed and Dimensions were the formal databases; Consensus and SciSpace served as supplementary discovery aids. Two reviewers screened records and full texts; effects were summarized as Hedges' g using a random-effects model, restricted maximum likelihood estimation of between-study variance, and Hartung–Knapp–Sidik–Jonkman confidence intervals. Outcomes were analyzed separately, with an adapted OHAT assessment of experimental bias and a qualified assessment of certainty.

Status: The article reports a final search on 22 November 2025. This document records and clarifies its methods retrospectively and does not report the review findings.

Introduction

Iron oxide nanoparticles can make mesenchymal stromal cells, or matrices carrying them, responsive to magnetic stimulation. Such systems may affect osteogenic markers through mechanical, biochemical, or thermal pathways. The interventions are diverse: static, time-varying, and pulsed fields are physically distinct, while particle dose, coating, localization, and cell source can modify their action. Pooling results therefore requires an explicit comparison and careful interpretation of heterogeneity.

The supplied Research Square example is a protocol for a scoping review and uses a mapping question and narrative synthesis [1]. This review instead estimates intervention effects, assesses experimental bias, and

performs a meta-analysis. Its structure follows the example's method-article format while drawing protocol items from PRISMA-P [2] and analysis methods from the Cochrane Handbook [3].

Review question and objectives

Primary question: Among mesenchymal stromal cells associated with iron oxide magnetic nanoparticles, what is the effect of external magnetic-field exposure on osteogenic differentiation compared with otherwise matched cells exposed to the nanoparticles but not to the field?

Secondary objectives are to characterize variation by field regime, magnetic flux density, nanoparticle characteristics, cell source, culture conditions, and assay; to summarize particle internalization and plausible mechanisms; and to describe animal bone-repair outcomes separately without treating them as equivalent to cell-culture markers.

Methods

Eligibility criteria

Element	Eligible evidence	Exclusions or synthesis restriction
Population	Primary mesenchymal stromal cells of human or animal origin; bone marrow, adipose tissue, dental pulp, umbilical tissues and other described sources.	Non-mesenchymal cell lines; cells whose identity or origin cannot be established.
Intervention	Iron oxide-based magnetic nanoparticles (including magnetite and maghemite), internalized by or otherwise associated with cells or a culture scaffold, with an external static, alternating, or pulsed magnetic field.	Non-iron-oxide or non-magnetic particles; nanoparticle exposure alone or field exposure alone as the intervention.
Comparator	For descriptive synthesis, a clearly described control without one or both intervention components.	For meta-analysis, no matched nanoparticle-exposed, field-free group; this comparator isolates the incremental field effect.
Outcomes	Measured osteogenic differentiation: alkaline phosphatase, mineralization, osteogenic transcripts or proteins.	No osteogenic endpoint; qualitative-only data may inform narrative synthesis but not a numerical effect estimate.
Design	Original controlled in vitro studies; studies with an animal component are eligible, with animal outcomes described separately.	Reviews, editorials, case reports, conference abstracts without usable full data, and purely clinical studies.
Reports	Full reports in English or Russian, with no publication-date restriction. Multiple reports of one experiment are linked.	Other languages if no reliable assessment or translation is possible; inaccessible full text after reasonable retrieval efforts.

The unit of inclusion is the independent experimental study, not the publication. Multiple papers describing overlapping experiments will be reconciled using sample, protocol, and outcome details. Iron oxide particles in a scaffold are eligible when an external field is applied and mesenchymal stromal-cell osteogenic outcomes are measured; such designs will be considered separately when their exposure is not comparable with intracellular labeling.

Information sources and search strategy

The supplied article reports formal searches in PubMed and Dimensions from database inception to 22 November 2025, without a date limit. Reference lists of eligible papers and relevant reviews were examined. Consensus and SciSpace were used as supplementary discovery platforms, with their results deduplicated and screened using the same criteria; their proprietary ranking does not make them substitutes for reproducible bibliographic searches. Exact original database exports and complete historical search strings were not present in the supplied files. Appendix 1 supplies a reproducible PubMed formulation for an update or verification; it must not be mislabeled as the exact query executed in 2025.

The search combines four concepts: mesenchymal stromal or stem cells; iron oxide magnetic nanoparticles; magnetic fields; and osteogenic differentiation. Syntax should be translated separately for Dimensions, with the full query, interface, date, record count, and export format archived. A published update should identify its own search date and report any addition of databases such as Scopus or Web of Science as an amendment.

Selection of studies

Records were deduplicated by DOI, PMID, and normalized title using a custom Python procedure and then managed in Zotero, as described in the manuscript. Two reviewers independently assessed titles and abstracts and then full texts against eligibility criteria. Differences were resolved by discussion. Reasons for full-text exclusion and record counts at each stage are to be shown in a PRISMA 2020 flow diagram [4]. Where a citation could not be obtained or was duplicated across multiple reports, the disposition is documented separately.

Data collection and outcome definitions

A standardized extraction form records citation, country, study design, species and MSC source, culture passage, biological replicate unit, particle core, coating, size and size-measurement method, concentration, incubation and localization, scaffold, osteogenic medium, field waveform, flux density, frequency, gradient when reported, exposure schedule, and follow-up. For each arm and endpoint it records units, time point, mean, standard deviation, independently replicated sample size, graph-derived values, and source location. Two reviewers independently check extracted outcome data and resolve discrepancies by discussion.

Nine quantitative outcome families are kept separate: alkaline phosphatase enzyme activity, alkaline phosphatase transcript, mineralization, RUNX2 transcript, osteocalcin transcript and protein, type I collagen transcript and protein, and osteopontin transcript. Distinct assays or molecular levels are not treated as identical. Direction is harmonized so a positive effect indicates more osteogenic differentiation in the combined exposure group. Viability, particle uptake and pathway measurements support descriptive mechanistic interpretation but are not substituted for an osteogenic endpoint.

Values plotted only in figures may be digitized after axis calibration, with a flag for data origin and a sensitivity check. Standard error is converted to standard deviation only when the replicate count is known. If numerical results or replicate counts are missing, the authors may be contacted. The manuscript reports assigning $n = 3$ when n was absent and testing exclusion of these imputed studies; such imputed n is flagged rather than represented as observed. Technical wells or images are not counted as independent biological replicates when they share the same culture preparation.

Selection among multiple results

Exactly one comparison per independent study per outcome enters a given conventional meta-analysis, to prevent repeated use of a shared control. The manuscript describes choosing the key time point for each marker (approximately day 7 for early alkaline phosphatase and transcript outcomes, and days 14–21 for mineralization and late markers), then the main reported non-toxic working particle dose (typically 10–50 μg/mL), and the principal magnetic regimen identified by the study authors. If several choices remain, the decision and rationale are logged without reference to effect magnitude. Alternate eligible arms and time points remain available for

narrative and sensitivity analyses. Independent experiments within one report can be included separately only when independence is established.

Assessment of risk of bias

Two reviewers evaluate cell-culture studies using prespecified operational questions adapted from OHAT [5]. Domains cover allocation of culture units; equivalence of conditions and personnel blinding where feasible; blinded outcome assessment; completeness of data; selective reporting; nanoparticle characterization and cytotoxicity assessment; and independent replication. Each domain receives definitely low, probably low, probably high, or definitely high risk, with supporting quotations. Missing reporting is distinguished from a demonstrated defect, although the supplied manuscript classified unreported safeguards conservatively as probably high risk. Characterization, viability testing and replicate reporting are additionally presented as methodological quality signals because they are not always direct bias domains. Animal outcomes are interpreted separately, and this retrospective record does not claim that a dedicated animal risk-of-bias tool was applied.

Synthesis and meta-analysis

A structured table summarizes all eligible experiments. Quantitative synthesis is limited to the same outcome family and the “nanoparticles plus field” versus “nanoparticles without field” comparison. Means, standard deviations and independent n are converted to the small-sample-corrected standardized mean difference, Hedges’ g, with 95% confidence intervals. Transcript and protein readouts are analyzed in distinct pools. If an outcome uses a fundamentally different construct or the designs are clinically or experimentally incomparable, results are described without pooling.

A random-effects model estimates between-study variance τ^2 by restricted maximum likelihood. Hartung–Knapp–Sidik–Jonkman intervals use a t distribution with $k-1$ degrees of freedom where applicable; with very few studies the limitations of this interval are explicitly noted. Cochran’s Q, I^2 and τ^2 describe statistical dispersion, alongside field type, particle formulation, and cell source. A 95% prediction interval is reported when meaningful and calculable. An I^2 above 75% triggers emphasis on subgroup patterns and narrative explanation; it is not the sole decision rule for whether studies can be combined. The overall pool across static, alternating and pulsed fields is explicitly exploratory, with interpretation centered on physically coherent strata [3].

Where data permit, subgroup analyses consider static versus time-varying/pulsed fields, flux density, MSC tissue source, and particle concentration or coating. Univariate meta-regression is exploratory and limited by the number of independent studies per covariate; correlations at the study level do not establish causal parameter effects. Sensitivity analyses exclude each study in turn, studies with imputed n, and extreme comparisons ($|g| > 10$); the manuscript also reports a log response-ratio analysis for outcomes with positive means. Funnel plots and Egger’s regression are considered only when at least ten independent comparisons inform one outcome, with asymmetry described as small-study effects rather than proof of publication bias. Trim-and-fill is an exploratory sensitivity analysis. The Holm procedure adjusts the family of nine primary outcome tests, with unadjusted and adjusted p values both reported.

If animal defect-repair measurements are available, bone-volume fraction, bone mineral density and related endpoints are presented narratively by model and endpoint. They are not pooled with in vitro ALP, mineralization, or molecular markers. Graph-derived values, imputed sample sizes and different exposure regimes are marked in all effect tables.

Certainty and interpretation

The certainty of each outcome is assessed with an explicitly adapted GRADE framework, considering risk of bias, inconsistency, indirectness, imprecision and suspected reporting bias [6]. Because GRADE was devised for clinical questions, any starting level assigned to controlled laboratory experiments is a review-specific convention rather than a validated equivalence with randomized clinical trials. Very large standardized effects in

small cell-culture experiments are not automatically upgraded. Results indicate laboratory marker changes and cannot alone establish improvement in human bone repair or an optimal clinical field regimen.

Protocol provenance and amendments

This document reconciles the supplied Russian-language draft protocol and the completed English manuscript. The following points require transparent reporting in any public protocol or article version:

Topic	Earlier draft	Method documented in manuscript
Comparator	Any no-particle/no-field, particles-only, or field-only control.	Meta-analysis restricted to particles-only, field-free controls; other controls support descriptive synthesis.
Sources	PubMed, Dimensions, SciSpace and Consensus listed together.	PubMed and Dimensions as formal databases; SciSpace and Consensus as supplementary discovery aids; final search 22 November 2025.
Multiplicity and sensitivity	General sensitivity and subgroup proposals.	One comparison per study/outcome; imputed n and extreme-effect sensitivity analyses; nine-outcome Holm adjustment.
Registration	No confirmed registration in the draft.	Manuscript includes an unfilled OSF registration DOI and calls registration retrospective; no identifier is asserted here.

The timing of each substantive change relative to screening and analysis cannot be verified from the supplied files. For publication, retain dated protocol versions, original exports, screening decisions, and analysis code so that prespecified decisions can be distinguished from later exploratory decisions. This record should be cited as retrospective methodological documentation, not as a prospectively registered protocol.

Declarations

Funding: The associated review manuscript attributes support to the state assignment of the Ministry of Health of the Russian Federation No. 1024022100092-5. The authors should confirm that this funding statement also applies to the protocol.

Conflicts of interest: The associated manuscript declares no conflicts of interest; this statement should be confirmed by all protocol authors before public submission.

Ethics: The review uses published experimental reports; no new work with human participants or animals is described in this protocol.

Data and code: The manuscript states that extracted data and analysis code are available upon request. Public release of dated search strings, exports, selection decisions and analysis code would improve reproducibility.

Appendix 1 Reproducible search formulation

Proposed PubMed search for a verification or update. This string is reconstructed from the supplied topic and search concepts. The exact historical 2025 query was not supplied, so the record count and execution date must be entered from a new run and reported as an update.

```
(("Mesenchymal Stem Cells"[Mesh] OR mesenchymal stem cell*[tiab] OR mesenchymal stromal cell*[tiab] OR
bone marrow stromal cell*[tiab] OR adipose-derived stem cell*[tiab] OR dental pulp stem cell*[tiab])
AND ("Magnetite Nanoparticles"[Mesh] OR magnetic nanoparticle*[tiab] OR iron oxide nanoparticle*[tiab]
OR superparamagnetic iron oxide[tiab] OR SPION*[tiab] OR magnetite[tiab] OR maghemite[tiab] OR
```

```
Fe3O4[tiab]) AND ("Magnetic Fields"[Mesh] OR magnetic field*[tiab] OR electromagnetic field*[tiab] OR
static magnetic field*[tiab] OR pulsed electromagnetic field*[tiab] OR PEMF[tiab]) AND
("Osteogenesis"[Mesh] OR osteogenic[tiab] OR osteogenesis[tiab] OR bone differentiation[tiab] OR
alkaline phosphatase[tiab] OR mineralization[tiab] OR RUNX2[tiab] OR osteocalcin[tiab]))
```

For Dimensions, translate the four concept blocks to its supported title/abstract or full-data search syntax, save the exact executed string and interface settings, and export identifiers, abstracts, DOI and source metadata. For supplemental discovery platforms, archive the natural-language prompt, date, sorted results or saved exports, and deduplication method. No publication-date filter is applied in the reconstructed PubMed formulation.

References

1. Issa J, Abou Chaar M, Kempisty B, Mozdziak P, Dyszkiewicz-Konwińska M. Artificial intelligence-based imaging analysis of stem cells: a systematic scoping review protocol. Research Square. Posted 8 March 2022. doi:10.21203/rs.3.rs-1422776/v1.

2. Moher D, Shamseer L, Clarke M, et al. Preferred reporting items for systematic review and meta-analysis protocols (PRISMA-P) 2015 statement. Syst Rev. 2015;4:1. doi:10.1186/2046-4053-4-1.

3. Cochrane Handbook for Systematic Reviews of Interventions, version 6.5, Chapter 10: Analysing data and undertaking meta-analyses. https://www.cochrane.org/authors/handbooks-and-manuals/handbook/current/chapter-10

4. Page MJ, McKenzie JE, Bossuyt PM, et al. The PRISMA 2020 statement: an updated guideline for reporting systematic reviews. BMJ. 2021;372:n71. doi:10.1136/bmj.n71.

5. National Toxicology Program. OHAT Risk of Bias Tool. https://ntp.niehs.nih.gov/research/assessments/noncancer/riskbias

6. Zhang Y, Akl EA, Schünemann HJ. Using systematic reviews in guideline development: the GRADE approach. Res Synth Methods. 2019;10:312–329. doi:10.1002/jrsm.1313.
