# Supplementary material for: Effect of Combined Iron Oxide Magnetic Nanoparticles and Magnetic Field Exposure on Osteogenic Differentiation of Mesenchymal Stem Cells: A Systematic Review and Meta-Analysis
Source: Bioengineering (Basel). 2026 Sep 30;13(10):1143. doi: 10.3390/bioengineering13101143 (PMC13701717; doi:10.3390/bioengineering13101143)
Supplement: Supplementary file 1 [file bioengineering-13-01143-s001.zip › Table_S1_Search_queries.pdf]

Search queries for PubMed:

(
"Mesenchymal Stem Cells"[Mesh] OR "Stromal Cells"[Mesh] OR "Adipose-Derived Stem Cells"[Mesh] OR "Bone Marrow Stromal Cells"[Mesh] OR "Dental Pulp Stem Cells"[Mesh] OR "Wharton's Jelly Cells"[Mesh] OR "mesenchymal stem cell" OR "mesenchymal stromal cell" OR "multipotent stromal cell" OR MSC OR "adipose derived stem cell" OR ADSC OR "bone marrow stem cell" OR BMSC OR "dental pulp stem cell" OR DPSC OR "umbilical cord stem cell"
)
AND
(
"Magnetite Nanoparticles"[Mesh] OR "Ferrosoferric Oxide"[Mesh] OR "magnetic nanoparticle" OR "iron oxide" OR magnetite OR Fe3O4 OR SPION
)
AND
(
"Electromagnetic Fields"[Mesh] OR "Magnetic Fields"[Mesh] OR "static magnetic field" OR "pulsed electromagnetic field" OR "alternating magnetic field" OR "magnetic stimulation" OR SMF OR PEMF
)
AND
(
"Osteogenesis"[Mesh] OR "Osteogenic Differentiation" OR "Bone Differentiation" OR osteogenic OR osteogenesis OR "alkaline phosphatase" OR ALP OR "Alizarin Red" OR "Bone Nodules" OR Runx2 OR Osteocalcin OR Osterix OR Sp7 OR "Collagen I" OR COL1A1
)

Search queries for Dimensions:

(
"Mesenchymal Stem Cell" OR "Mesenchymal Stromal Cell" OR "Multipopotent Stromal Cells" OR MSC OR "Adipose-Derived Stem Cell" OR ADSC OR "Bone Marrow Stem Cell" OR BMSC OR "Dental Pulp Stem Cell" OR DPSC OR "Umbilical Cord Stem Cell" OR UC-MSC OR "Wharton's Jelly Cell")
AND
(
"magnetic nanoparticle" OR "iron oxide" OR magnetite OR Fe3O4 OR SPION
)
AND
(
"Magnetic Field" OR "Electromagnetic Field" OR "static magnetic field" OR SMF OR "pulsed electromagnetic field" OR PEMF OR "alternating magnetic field" OR "magnetic stimulation"
)
AND
(
Osteogenesis OR "Osteogenic Differentiation" OR "Bone Differentiation" OR "alkaline phosphatase" OR ALP OR "Alizarin Red" OR "Bone Nodule" OR "Bone Nodules" OR Runx2 OR Osteocalcin OR Osterix OR Sp7 OR "Collagen I" OR COL1A1
)

Search query for Consensus:

What magnetic fields (type, parameters) and magnetic nanoparticles (type, concentration) are used to induce osteogenic differentiation of mesenchymal stromal cells?

Search query for Scispace:

The Effect of Magnetic Fe_3O_4 Nanoparticles and Magnetic Fields on Osteogenic Differentiation of Mesenchymal Stem Cells (1980-2025)
