# Supplementary material for: Effect of Combined Iron Oxide Magnetic Nanoparticles and Magnetic Field Exposure on Osteogenic Differentiation of Mesenchymal Stem Cells: A Systematic Review and Meta-Analysis
Source: Bioengineering (Basel). 2026 Sep 30;13(10):1143. doi: 10.3390/bioengineering13101143 (PMC13701717; doi:10.3390/bioengineering13101143)
Supplement: Supplementary file 1 [file bioengineering-13-01143-s001.zip › Table_S3_Additional_sensitivity_analyses.pdf]

Supplementary Table S3. Additional sensitivity analyses

Outlier truncation excludes comparisons with |Hedges' g| > 10. Imputed-n sensitivity excludes studies with n imputed as 3. lnRR = natural log of the response ratio (random-effects REML / HKSJ).

S3a. Outlier-truncated sensitivity (|SMD| > 10 excluded)

Outcome	k full	k trunc.	SMD	95% CI	p	I^2	Dropped studies
ALP activity	14	11	+2.05	[+0.31; +3.80]	0.025	73%	Estévez 2024, Estévez 2025, Russo 2020
ALP mRNA	8	7	+1.73	[-0.46; +3.92]	0.101	74%	Shuai 2020
Mineralization	7	6	+2.04	[+0.18; +3.91]	0.037	56%	Estévez 2025
Runx2 mRNA	8	7	+1.46	[-1.92; +4.85]	0.331	82%	Shuai 2020
Osteocalcin mRNA	4	4	+2.90	[-3.18; +8.98]	0.226	85%	—
Osteocalcin protein	3	2	+4.19	[-7.21; +15.58]	0.134	0%	Jiang 2016
COL1 mRNA	7	7	+1.73	[-0.31; +3.77]	0.083	74%	—
COL1 protein	2	2	+4.94	[-15.37; +25.24]	0.199	34%	—
Osteopontin mRNA	5	5	+1.39	[-2.22; +5.00]	0.346	79%	—

S3b. Sensitivity excluding studies with imputed n = 3

Outcome	k with	SMD with	k without	SMD without	95% CI without	p without	Imputed studies
ALP activity	14	+4.42	9	+5.63	[+0.15; +11.12]	0.045	Estévez 2025, Huang 2017, Khalili 2022, Kim 2015, Wang 2016
ALP mRNA	8	+2.87	5	+3.33	[-0.49; +7.14]	0.073	Estévez 2025, Lin 2019, Schäfer 2010
Mineralization	7	+2.41	5	+1.67	[+0.11; +3.23]	0.041	Estévez 2025, Khalili 2022
Runx2 mRNA	8	+3.09	7	+2.49	[-3.34; +8.33]	0.336	Khalili 2022
Osteocalcin mRNA	4	+2.90	3	+1.91	[-7.04; +10.85]	0.456	Khalili 2022
Osteocalcin protein	3	+4.47	2	+8.77	[-64.43; +81.96]	0.370	Estévez 2025
COL1 mRNA	7	+1.73	6	+2.12	[+0.40; +3.84]	0.025	Khalili 2022
COL1 protein	2	+4.94	2	+4.94	[-15.37; +25.24]	0.199	—
Osteopontin mRNA	5	+1.39	4	+2.18	[-1.75; +6.10]	0.176	Wang 2016

S3c. Alternative effect measure: ln(response ratio)

Outcome	k	lnRR	95% CI	p	I^2
ALP activity	14	+0.415	[+0.151; +0.678]	0.005	99%
ALP mRNA	8	+0.307	[+0.075; +0.539]	0.017	95%
Mineralization	7	+0.262	[+0.119; +0.406]	0.004	85%
Runx2 mRNA	8	+0.415	[-0.068; +0.898]	0.082	99%
Osteocalcin mRNA	4	+0.585	[-0.863; +2.034]	0.289	98%
Osteocalcin protein	3	+0.737	[-1.009; +2.483]	0.211	100%
COL1 mRNA	7	+0.254	[-0.043; +0.551]	0.082	92%
COL1 protein	2	+1.031	[-6.698; +8.761]	0.339	99%
Osteopontin mRNA	5	+0.203	[-0.218; +0.624]	0.251	95%
